# DNA methylation cooperates with genomic alterations during non-small cell lung cancer evolution

**DOI:** 10.1038/s41588-025-02307-x

**Published:** 2025-09-10

**Authors:** Francisco Gimeno-Valiente, Carla Castignani, Elizabeth Larose Cadieux, Nana E. Mensah, Xiaohong Liu, Kezhong Chen, Olga Chervova, Takahiro Karasaki, Clare E. Weeden, Corentin Richard, Siqi Lai, Carlos Martínez-Ruiz, Emilia L. Lim, Alexander M. Frankell, Thomas B. K. Watkins, Georgia Stavrou, Ieva Usaite, Wei-Ting Lu, Daniele Marinelli, Sadegh Saghafinia, Gareth A. Wilson, Pawan Dhami, Heli Vaikkinen, Jonathan Steif, Selvaraju Veeriah, Robert E. Hynds, Martin Hirst, Crispin Hiley, Andrew Feber, Özgen Deniz, Mariam Jamal-Hanjani, Nicholas McGranahan, TRACERx Consortium, Francisco Gimeno-Valiente, Carla Castignani, Elizabeth Larose Cadieux, Nana E. Mensah, Takahiro Karasaki, Clare E. Weeden, Corentin Richard, Carlos Martínez-Ruiz, Thomas B. K. Watkins, Georgia Stavrou, Ieva Usaite, Wei-Ting Lu, Sadegh Saghafinia, Gareth A. Wilson, Selvaraju Veeriah, Crispin Hiley, Mariam Jamal-Hanjani, Nicholas McGranahan, Robert Bentham, Thomas P. Jones, James R. M. Black, Michelle Dietzen, Maria Litovchenko, Kerstin Thol, Abigail Bunkum, Sonya Hessey, Wing Kin Liu, Nicolai J. Birkbak, Ariana Huebner, Clare Puttick, David A. Moore, Dhruva Biswas, Kristiana Grigoriadis, Maise Al Bakir, Olivia Lucas, Roberto Vendramin, Sophia Ward, Sian Harries, Simone Zaccaria, Rija Zaidi, Lucrezia Patruno, Despoina Karagianni, Sergio A. Quezada, Supreet Kaur Bola, Martin D. Forster, Siow Ming Lee, Cristina Naceur-Lombardelli, Krupa Thakkar, Monica Sivakumar, Sharon Vanloo, Antonia Toncheva, Paulina Prymas, Bushra Mussa, Michalina Magala, Elizabeth Keene, Michelle M. Leung, Jeanette Kittel, Kerstin Haase, Kexin Koh, Rachel Scott, Chris Bailey, Oriol Pich, Rachel Rosenthal, Andrew Rowan, Claudia Lee, Emma Colliver, Katey S. S. Enfield, Mihaela Angelova, Cian Murphy, Maria Zagorulya, Jayant K. Rane, Zhihui Zhang, Sarah Benafif, Dionysis Papadatos-Pastos, James Wilson, Tanya Ahmad, Teresa Marafioti, Elaine Borg, Mary Falzon, Reena Khiroya, Yien Ning Sophia Wong, Emilie Martinoni Hoogenboom, Fleur Monk, James W. Holding, Junaid Choudhary, Kunal Bhakhri, Pat Gorman, Robert C. M. Stephens, Maria Chiara Pisciella, Steve Bandula, Jerome Nicod, Angela Dwornik, Angeliki Karamani, Benny Chain, David R. Pearce, Gerasimos-Theodoros Mastrokalos, Helen L. Lowe, James L. Reading, John A. Hartley, Kayalvizhi Selvaraju, Leah Ensell, Mansi Shah, Piotr Pawlik, Samuel Gamble, Seng Kuong Anakin Ung, Victoria Spanswick, Yin Wu, Jason F. Lester, Sean Dulloo, Dean A. Fennell, Amrita Bajaj, Apostolos Nakas, Azmina Sodha-Ramdeen, Mohamad Tufail, Molly Scotland, Rebecca Boyles, Sridhar Rathinam, Claire Wilson, Gurdeep Matharu, Jacqui A. Shaw, Ekaterini Boleti, Heather Cheyne, Mohammed Khalil, Shirley Richardson, Tracey Cruickshank, Gillian Price, Keith M. Kerr, Jack French, Kayleigh Gilbert, Babu Naidu, Akshay J. Patel, Gary Middleton, Aya Osman, Mandeesh Sangha, Gerald Langman, Helen Shackleford, Madava Djearaman, Angela Leek, Jack Davies Hodgkinson, Nicola Totton, Philip Crosbie, Eustace Fontaine, Felice Granato, Juliette Novasio, Kendadai Rammohan, Leena Joseph, Paul Bishop, Vijay Joshi, Sara Waplington, Adam Atkin, Katherine D. Brown, Mathew Carter, Anshuman Chaturvedi, Pedro Oliveira, Colin R. Lindsay, Fiona H. Blackhall, Yvonne Summers, Jonathan Tugwood, Caroline Dive, Matthew G. Krebs, Antonio Paiva-Correia, Hugo J. W. L. Aerts, Roland F. Schwarz, Tom L. Kaufmann, Zoltan Szallasi, Miklos Diossy, Roberto Salgado, George Kassiotis, Imran Noorani, Eva Grönroos, Jacki Goldman, Mickael Escudero, Philip Hobson, Stefan Boeing, Tamara Denner, Vittorio Barbè, William Hill, Yutaka Naito, Erik Sahai, Zoe Ramsden, Emma Nye, Richard Kevin Stone, Karl S. Peggs, Catarina Veiga, Gary Royle, Charles-Antoine Collins-Fekete, Francesco Fraioli, Paul Ashford, Arjun Nair, Alexander James Procter, Asia Ahmed, Magali N. Taylor, David Lawrence, Davide Patrini, Neal Navani, Ricky M. Thakrar, Sam M. Janes, Zoltan Kaplar, Allan Hackshaw, Camilla Pilotti, Rachel Leslie, Anne-Marie Hacker, Sean Smith, Aoife Walker, Anca Grapa, Hanyun Zhang, Khalid AbdulJabbar, Xiaoxi Pan, Yinyin Yuan, David Chuter, Mairead MacKenzie, Serena Chee, Patricia Georg, Aiman Alzetani, Judith Cave, Eric Lim, Andrew G. Nicholson, Paulo De Sousa, Simon Jordan, Alexandra Rice, Hilgardt Raubenheimer, Harshil Bhayani, Lyn Ambrose, Anand Devaraj, Hema Chavan, Sofina Begum, Silviu I. Buderi, Daniel Kaniu, Mpho Malima, Sarah Booth, Nadia Fernandes, Pratibha Shah, Chiara Proli, Madeleine Hewish, Sarah Danson, Michael J. Shackcloth, Lily Robinson, Peter Russell, Kevin G. Blyth, Andrew Kidd, Craig Dick, John Le Quesne, Alan Kirk, Mo Asif, Rocco Bilancia, Nikos Kostoulas, Jennifer Whiteley, Mathew Thomas, Stephan Beck, Jonas Demeulemeester, Charles Swanton, Peter Van Loo, Nnennaya Kanu, Stephan Beck, Jonas Demeulemeester, Miljana Tanić, Charles Swanton, Peter Van Loo, Nnennaya Kanu

**Affiliations:** 1 Cancer Research UK Lung Cancer Centre of Excellence, University College London Cancer Institute, London, UK; 2 Cancer Genomics Laboratory, The Francis Crick Institute, London, UK; 3 Medical Genomics, University College London Cancer Institute, London, UK; 4 Cancer Evolution and Genome Instability Laboratory, The Francis Crick Institute, London, UK; 5 Thoracic Oncology Institute, Peking University People’s Hospital, Beijing, China; 6 Department of Thoracic Surgery, Peking University People’s Hospital, Beijing, China; 7 Cancer Metastasis Laboratory, University College London Cancer Institute, London, UK; 8 Department of Genetics, The University of Texas MD Anderson Cancer Center, Houston, TX USA; 9 Cancer Genome Evolution Research Group, Cancer Research UK Lung Cancer Centre of Excellence, University College London Cancer Institute, London, UK; 10 Department of Experimental Medicine, Sapienza University, Rome, Italy; 11 Genomics Research Platform, R&D Department, Guy’s and St Thomas’ NHS Foundation Trust, London, UK; 12 Department of Microbiology and Immunology, Michael Smith Laboratories, University of British Columbia, Vancouver, British Columbia Canada; 13 Canada’s Michael Smith Genome Sciences Centre, BC Cancer, Vancouver, British Columbia Canada; 14 Division of Surgery and Interventional Science Medical Genomics, University College London, London, UK; 15 Centre for Molecular Pathology, Royal Marsden Hospital Trust, London, UK; 16 Translational Epigenetic, Molecular Pathology, The Institute of Cancer Research, London, UK; 17 Centre for Haemato-Oncology, Barts Cancer Institute, Queen Mary University of London, London, UK; 18 QMUL Centre for Epigenetics, London, UK; 19 Department of Medical Oncology, University College London Hospitals, London, UK; 20 VIB–KU Leuven Center for Cancer Biology, Leuven, Belgium; 21 Integrative Cancer Genomics Laboratory, Department of Oncology, KU Leuven, Leuven, Belgium; 22 Experimental Oncology, Institute for Oncology and Radiology of Serbia, Belgrade, Serbia; 23 Department of Genomic Medicine, The University of Texas MD Anderson Cancer Center, Houston, TX USA; 24 Computational Cancer Genomics Research Group, University College London Cancer Institute, London, UK; 25 Department of Molecular Medicine, Aarhus University Hospital, Aarhus, Denmark; 26 Department of Clinical Medicine, Aarhus University, Aarhus, Denmark; 27 Bioinformatics Research Centre, Aarhus University, Aarhus, Denmark; 28 Department of Cellular Pathology, University College London Hospitals, London, UK; 29 Bill Lyons Informatics Centre, University College London Cancer Institute, London, UK; 30 University College London Hospitals, London, UK; 31 Tumour Immunogenomics and Immunosurveillance Laboratory, University College London Cancer Institute, London, UK; 32 Genomics Science Technology Platform, The Francis Crick Institute, London, UK; 33 Immune Regulation and Tumour Immunotherapy Group, Cancer Immunology Unit, Research Department of Haematology, University College London Cancer Institute, London, UK; 34 University College London Cancer Institute, London, UK; 35 The Whittington Hospital NHS Trust, London, UK; 36 National Cancer Centre, Singapore City, Singapore; 37 Singleton Hospital, Swansea Bay University Health Board, Swansea, UK; 38 University of Leicester, Leicester, UK; 39 University Hospitals of Leicester NHS Trust, Leicester, UK; 40 Leicester Medical School, University of Leicester, Leicester, UK; 41 Cancer Research Centre, University of Leicester, Leicester, UK; 42 Royal Free London NHS Foundation Trust, London, UK; 43 Aberdeen Royal Infirmary NHS Grampian, Aberdeen, UK; 44 Department of Medical Oncology, Aberdeen Royal Infirmary NHS Grampian, Aberdeen, UK; 45 University of Aberdeen, Aberdeen, UK; 46 Department of Pathology, Aberdeen Royal Infirmary NHS Grampian, Aberdeen, UK; 47 Birmingham Acute Care Research Group, Institute of Inflammation and Ageing, University of Birmingham, Birmingham, UK; 48 Guy’s and St Thomas’ NHS Foundation Trust, London, UK; 49 University Hospital Birmingham NHS Foundation Trust, Birmingham, UK; 50 Institute of Immunology and Immunotherapy, University of Birmingham, Birmingham, UK; 51 Manchester Cancer Research Centre Biobank, Manchester, UK; 52 Wythenshawe Hospital, Manchester University NHS Foundation Trust, Manchester, UK; 53 Division of Infection, Immunity and Respiratory Medicine, University of Manchester, Manchester, UK; 54 Cancer Research UK Lung Cancer Centre of Excellence, University of Manchester, Manchester, UK; 55 The Christie NHS Foundation Trust, Manchester, UK; 56 Division of Cancer Sciences, The University of Manchester and The Christie NHS Foundation Trust, Manchester, UK; 57 CRUK Manchester Institute Cancer Biomarker Centre, University of Manchester, Manchester, UK; 58 Manchester University NHS Foundation Trust, Manchester, UK; 59 Artificial Intelligence in Medicine (AIM) Program, Mass General Brigham, Harvard Medical School, Boston, MA USA; 60 Department of Radiation Oncology, Brigham and Women’s Hospital, Dana–Farber Cancer Institute, Harvard Medical School, Boston, MA USA; 61 Radiology and Nuclear Medicine, CARIM & GROW, Maastricht University, Maastricht, the Netherlands; 62 Institute for Computational Cancer Biology, Center for Integrated Oncology (CIO), Cancer Research Center Cologne Essen (CCCE), Faculty of Medicine and University Hospital Cologne, University of Cologne, Cologne, Germany; 63 Berlin Institute for the Foundations of Learning and Data (BIFOLD), Berlin, Germany; 64 Berlin Institute for Medical Systems Biology, Max Delbrück Center for Molecular Medicine in the Helmholtz Association (MDC), Berlin, Germany; 65 Danish Cancer Institute, Copenhagen, Denmark; 66 Computational Health Informatics Program, Boston Children’s Hospital, Boston, MA USA; 67 Department of Bioinformatics, Semmelweis University, Budapest, Hungary; 68 Department of Physics of Complex Systems, ELTE Eötvös Loránd University, Budapest, Hungary; 69 Department of Pathology, ZAS Hospitals, Antwerp, Belgium; 70 Division of Research, Peter MacCallum Cancer Centre, Melbourne, Victoria, Australia; 71 The Francis Crick Institute, London, UK; 72 Department of Infectious Disease, Faculty of Medicine, Imperial College London, London, UK; 73 Department of Neurosurgery, National Hospital for Neurology and Neurosurgery, London, UK; 74 Institute of Neurology, University College London, London, UK; 75 Experimental Histopathology, The Francis Crick Institute, London, UK; 76 Department of Haematology, University College London Hospitals, London, UK; 77 Cancer Immunology Unit, Research Department of Haematology, University College London Cancer Institute, London, UK; 78 Centre for Medical Image Computing, Department of Medical Physics and Biomedical Engineering, London, UK; 79 Department of Medical Physics and Bioengineering, University College London Cancer Institute, London, UK; 80 Institute of Nuclear Medicine, Division of Medicine, University College London, London, UK; 81 Institute of Structural and Molecular Biology, University College London, London, UK; 82 Department of Radiology, University College London Hospitals, London, UK; 83 UCL Respiratory, Department of Medicine, University College London, London, UK; 84 Department of Thoracic Surgery, University College London Hospital NHS Trust, London, UK; 85 Department of Thoracic Medicine, University College London Hospitals, London, UK; 86 Lungs for Living Research Centre, UCL Respiratory, Department of Medicine, University College London, London, UK; 87 Integrated Radiology Department, North-Buda St John’s Central Hospital, Budapest, Hungary; 88 Institute of Nuclear Medicine, University College London Hospitals, London, UK; 89 Cancer Research UK & UCL Cancer Trials Centre, London, UK; 90 The Institute of Cancer Research, London, UK; 91 Garvan Institute of Medical Research, Sydney, New South Wales Australia; 92 Case45, London, UK; 93 The University of Texas MD Anderson Cancer Center, Houston, TX USA; 94 Independent Cancer Patient’s Voice, London, UK; 95 University Hospital Southampton NHS Foundation Trust, Southampton, UK; 96 The NIHR Southampton Biomedical Research Centre, University Hospital Southampton NHS Foundation Trust, Southampton, UK; 97 Department of Oncology, University Hospital Southampton NHS Foundation Trust, Southampton, UK; 98 Academic Division of Thoracic Surgery, Imperial College London, London, UK; 99 Royal Brompton and Harefield Hospitals, Part of Guy’s and St Thomas’ NHS Foundation Trust, London, UK; 100 National Heart and Lung Institute, Imperial College, London, UK; 101 Royal Surrey Hospital, Royal Surrey Hospitals NHS Foundation Trust, Guildford, UK; 102 University of Surrey, Guildford, UK; 103 University of Sheffield, Sheffield, UK; 104 Sheffield Teaching Hospitals NHS Foundation Trust, Sheffield, UK; 105 Liverpool Heart and Chest Hospital, Liverpool, UK; 106 Princess Alexandra Hospital, The Princess Alexandra Hospital NHS Trust, Harlow, UK; 107 School of Cancer Sciences, University of Glasgow, Glasgow, UK; 108 Beatson Institute for Cancer Research, University of Glasgow, Glasgow, UK; 109 Queen Elizabeth University Hospital, Glasgow, UK; 110 Institute of Infection, Immunity & Inflammation, University of Glasgow, Glasgow, UK; 111 NHS Greater Glasgow and Clyde, Glasgow, UK; 112 Cancer Research UK Scotland Institute, Glasgow, UK; 113 Institute of Cancer Sciences, University of Glasgow, Glasgow, UK; 114 NHS Greater Glasgow and Clyde Pathology Department, Queen Elizabeth University Hospital, Glasgow, UK; 115 Golden Jubilee National Hospital, Clydebank, UK

**Keywords:** Non-small-cell lung cancer, Epigenetics

## Abstract

Aberrant DNA methylation has been described in nearly all human cancers, yet its interplay with genomic alterations during tumor evolution is poorly understood. To explore this, we performed reduced representation bisulfite sequencing on 217 tumor and matched normal regions from 59 patients with non-small cell lung cancer from the TRACERx study to deconvolve tumor methylation. We developed two metrics for integrative evolutionary analysis with DNA and RNA sequencing data. Intratumoral methylation distance quantifies intratumor DNA methylation heterogeneity. M_R_/M_N_ classifies genes based on the rate of hypermethylation at regulatory (M_R_) versus nonregulatory (M_N_) CpGs to identify driver genes exhibiting recurrent functional hypermethylation. We identified DNA methylation-linked dosage compensation of essential genes co-amplified with neighboring oncogenes. We propose two complementary mechanisms that converge for copy number alteration-affected chromatin to undergo the epigenetic equivalent of an allosteric activity transition. Hypermethylated driver genes under positive selection may open avenues for therapeutic stratification of patients.

## Abstract (web summary)

Integrated multi-omic analyses using samples from the TRACERx study highlight cross-talk between DNA hypermethylation and genomic lesions in non-small cell lung cancer.

## Main

Lung cancer, of which the predominant group is non-small cell lung cancer (NSCLC), is the leading cause of cancer-related death worldwide^1^. Genomic and transcriptomic studies of the two major NSCLC subgroups, lung adenocarcinoma (LUAD) and lung squamous cell carcinoma (LUSC), have provided a deep understanding of the evolutionary processes that provide subclones with selective advantages, through the accumulation of genetic driver events^2–4^.

Recent studies highlighted evidence of non-genomic evolution in cancer development, neoantigen silencing^5^ and acquired therapeutic resistance^6,7^. An important proportion of these resistance mechanisms are driven by epigenetic alterations, including DNA methylation.

Distinguishing DNA methylation events that play a causative role in cancer evolution from innocuous passenger events is not trivial^8,9^. Recent algorithms for driver gene discovery incorporate biological features known to affect the rate of stochastic DNA methylation changes and have identified genes known to affect progression-free survival^10–13^. Although these approaches have been useful for identifying candidate DNA methylation cancer genes, they often do not incorporate the selection of hypermethylation events with functional impact and may inadvertently also identify neutral passengers. Analogous approaches to the implementation of the nonsynonymous-to-synonymous mutations ratio (dN/dS) in evolutionary genetics with covariates for the identification of single-nucleotide variant (SNV) driver events^14^ may enable genuine DNA methylation drivers to be distinguished from neutral passenger events.

Many approaches have been developed for methylome profiling, most of which require either array hybridization or sequencing of bisulfite-converted DNA^15,16^. However, the varying purities of bulk solid tumor samples and the high degree of copy number (CN) instability associated with lung cancer, confound tumor methylation rates^17^. To overcome these limitations, we recently developed Copy number-Aware Methylation Deconvolution Analysis of Cancers (CAMDAC), which models the pure tumor methylation rate as the difference between the methylation rate in the bulk tumor and normal contaminants weighted for tumor CN and purity^17^. We applied CAMDAC to the multi-region tumor sampling and longitudinal lung TRAcking Cancer Evolution through therapy/Rx (TRACERx) study. Through an integrative analysis with gene expression and whole-exome sequencing (WES), we uncover the interplay between DNA hypermethylation and genomic alterations in NSCLC drawing on the concept of allostery^18^.

## Results

### The cancer cell-specific DNA methylation landscape of NSCLC

To elucidate the roles of DNA methylation during NSCLC evolution, we performed reduced representation bisulfite sequencing (RRBS) on 217 tumor regions and 59 paired normal adjacent tissues (NATs) from 59 patients in the TRACERx cohort (Supplementary Fig. 1a–d).

**Fig. 1:**
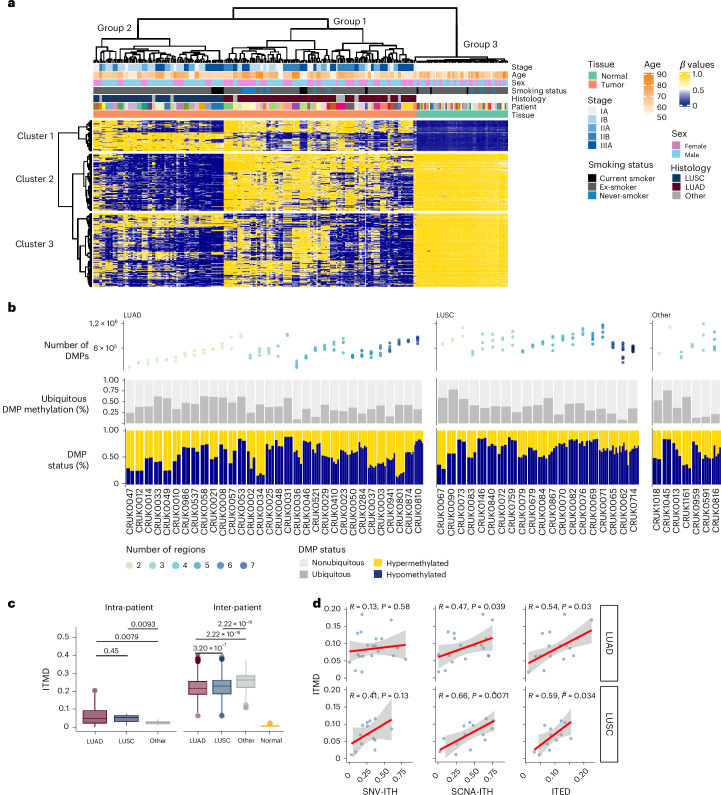
Global DNA methylation landscape in the TRACERx lung cancer study. **a**, Unsupervised hierarchical clustering of the 5,000 most variable CpGs in 217 tumor regions from 59 patients and 59 matched NAT samples. Yellow, hypermethylated CpGs; blue, hypomethylated CpGs. Groups correspond to patient samples and clusters correspond to CpGs. **b**, The number of DMPs, the percentage of ubiquitous DMPs (fraction of regions in which the DMP is present) and the methylation status of the DMPs are illustrated, indicating the degree of ITH. Samples are stratified according to histological subtypes and arranged in ascending order from left to right based on the number of regions sampled. **c**, ITMD metric calculated across regions within (intra) and between (inter) tumors. The box plot shows the median, interquartile range (IQR) (Q1–Q3), whiskers extending to 1.5 times the IQR and outliers beyond this range (Wilcoxon rank-sum test). **d**, Correlations between ITMD score and other heterogeneity metrics; mutation (SNV-ITH), SCNA-ITH and ITED, depicted from left to right, for LUAD (top) and LUSC (bottom). The fitted line represents a smoothed trend estimated using a robust linear regression, with the shaded region indicating the 95% confidence interval.

**Extended Data Fig. 1:**
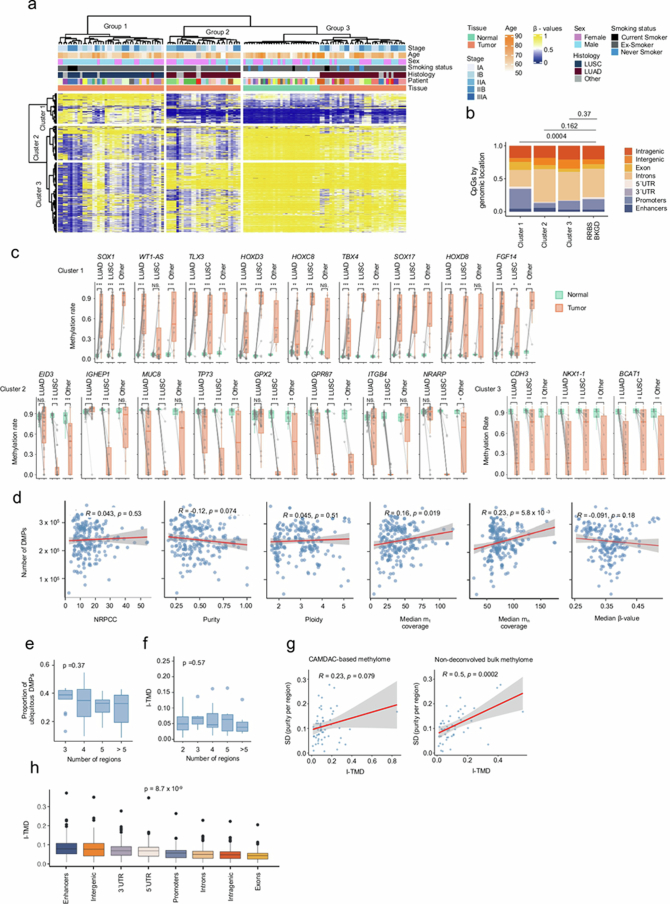
Global DNA methylation landscape in the TRACERx lung cancer study. **a**) Unsupervised hierarchical clustering of the 5,000 most variable CpGs in the bulk DNA methylation data. Yellow, hypermethylated CpGs, blue, hypomethylated CpGs. Groups correspond to patient samples and clusters correspond to CpGs. **b**) Genomic features representation of the 5,000 most variable CpGs identified using CAMDAC in the three clusters and in the background of CpGs in RRBS capture regions **c**) Methylation rate of CpGs in Clusters 1, 2 and 3, corresponding to promoter regions of genes in tumor and normal, classified by subtype from left to right: LUAD, LUSC, and other subtypes. Wilcoxon test, *P* < 0.001 (***), *P* < 0.01 (**), *P* < 0.05 (*). **d**) Correlation between the number of differentially methylated positions (DMPs) and the number of reads per chromosomal copy (NRPCC), purity, ploidy, median CpG coverage in the tumor and normal samples and median β-value. Median m_t_ and m_n_ coverage correspond to the number of reads per CpG in the CAMDAC-deconvolved and normal data respectively (Pearson’s correlation test). The fitted line represents a smoothed trend estimated using a robust linear regression (RLM), with the shaded region indicating the 95% confidence interval. **e**) Proportion of ubiquitous DMPs with respect to the number of regions sampled (ANOVA test). **f**) Relationship between ITMD value and the number of regions sampled (ANOVA test). The boxplot shows the median, interquartile range (Q1–Q3), whiskers extending to 1.5×IQR, and outliers beyond this range. **g**) Correlation between the standard deviation (SD) of purities across regions from the same patient tumor versus CAMDAC-based methylomes (left) and nondeconvolved bulk methylomes (right) ITMD (Pearson’s correlation test). The fitted line represents a smoothed trend estimated using a robust linear regression (RLM), with the shaded region indicating the 95% confidence interval. **h**) Relationship between ITMD value and the genomic feature annotation. ANOVA test, *P* < 0.001 (***). The boxplot shows the median, interquartile range (Q1–Q3), whiskers extending to 1.5×IQR, and outliers beyond this range.

Unsupervised hierarchical clustering using the 5,000 most variable CpGs based on CAMDAC methylation rates revealed three main groups of samples, largely corresponding to NAT, LUAD and LUSC (bootstrap probability value 98%; cluster stability values 0.98, 0.91 and 0.94, respectively; Fig. 1a and Methods), with most tumor regions clustering according to patient. Three clusters of CpG sites with distinct profiles were observed, regardless of the number of CpGs analyzed (Fowlkes–Mallows index > 0.96) (Fig. 1a). These profiles were not apparent using nondeconvolved bulk methylomes (Extended Data Fig. 1a). Cluster 1 was enriched in two subclusters of promoter CpGs found unmethylated in normal tissue and methylated in tumor samples, independent of histology (Fig. 1a and Extended Data Fig. 1b). This cluster was enriched in genes involved in differentiation and developmental processes (for example, *SOX1* and *SOX9, HOXD3* and *HOXD8*, and *TBX4*) and genes implicated as tumor suppressors (for example, *SOX1* and *SOX17, TSHZ3, WT1-AS*, and *FGF14*) (Extended Data Fig. 1c and Supplementary Tables 1 and 2). Clusters 2 and 3 captured CpG sites hypomethylated in the tumor. While cluster 2 was enriched in LUSC-specific hypomethylation, cluster 3 exhibited cohort-wide hypomethylation, with a small subset of CpGs selectively hypomethylated in LUAD (Fig. 1a and Supplementary Tables 3 and 4). Upon considering all promoter CpGs in principal component analyses, histological subtype was the sole clinical variable distinguishing tumors (Supplementary Fig. 2).

**Extended Data Fig. 2:**
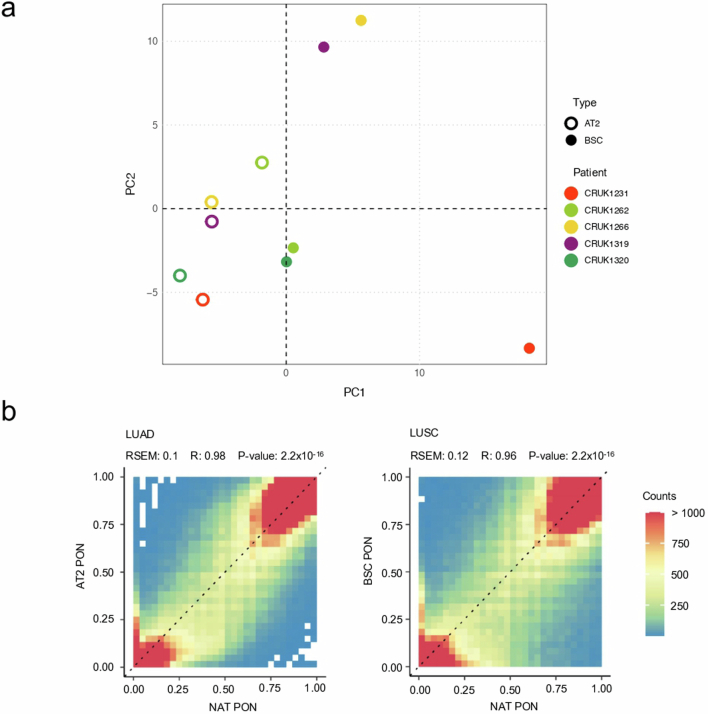
Analysis and characterization of cells of origin for LUAD and LUSC compared to normal adjacent tissue. **a**) Principal component analysis based on known transcriptomic signatures of cells-of-origin for LUAD (AT2) and LUSC (BSC). Freshly isolated populations were obtained via flow cytometry from five normal-adjacent tissue samples from the TRACERx cohort. **b**) Correlation of the β-values of a random set of 1 million CpGs (minimum coverage of 10 reads) between the panel of normal (PON) from the FACS sorted cells-of-origin (*y* axis) and the PON from NAT (*x* axis). AT2 PON versus NAT PON (LUAD, left) and BSC PON versus NAT PON (LUSC, right). Color scale (count) corresponds to the number of CpGs with overlapping methylation rates in both PONs.

To further characterize the tumor methylome, we next identified differentially methylated positions (DMPs) between tumor and normal samples using cancer-cell-specific methylation rates. To establish that bulk NAT serves as a reliable reference regardless of tumor subtype, we freshly isolated alveolar type 2 (AT2) cells, the cell of origin of LUAD, and basal cells (BSC), the cell of origin of LUSC, from bulk NAT from five TRACERx samples (Extended Data Fig. 2a); no significant differences were found in the methylation *β* values compared to bulk NAT (Extended Data Fig. 2b).

Proceeding with bulk patient-matched NAT, the median number of DMPs per sample varied between 48,080 and 362,775 (Fig. 1b); in the coverage range of our samples, it was robust to the number of reads per chromosomal copy^19^, purity and ploidy. Additionally, as expected, we observed a correlation with the average breadth of coverage, representing the number of reads covered in the tumor-normal pairs (Extended Data Fig. 1d). At the tumor level, a high variability in the proportion of DMPs shared ubiquitously by all regions was apparent (ranging from 0.09 to 0.78), which was not affected by the number of regions sampled per tumor (Extended Data Fig. 1e). In addition, the methylation status of DMPs showed high variability between tumors but limited variability between regions from the same tumor (Fig. 1b).

To further quantify the extent of DNA methylation heterogeneity, we computed intratumoral methylation distances (ITMDs) based on the pairwise Pearson distance between methylation rates at all CpGs across all sampled regions and across different tumors (Methods). The ITMD score was robust to the number of regions sampled (Extended Data Fig. 1f) and exhibited no association with purity after deconvolution with CAMDAC (Extended Data Fig. 1g). Compared to normal samples, tumors exhibited a 25-fold increase in inter-patient heterogeneity, indicating aberrant DNA methylation dynamics in tumors (Fig. 1c). In addition, inter-patient variability was higher than intra-patient variability across both histological subtypes (Fig. 1c). Intergenic and enhancer regions showed the highest variability, while promoter regions had significantly lower methylation heterogeneity, suggesting tighter regulation in promoter regions (Extended Data Fig. 1h).

Given the extensive genomic and transcriptomic intratumor heterogeneity (ITH) captured by TRACERx^2,3^, we next explored the interplay between epigenetic and genetic heterogeneity. The ITMD scores weakly correlated with mutation heterogeneity (SNV-ITH: LUAD, *R* = 0.13, *P* = 0.58; LUSC, *R* = 0.41, *P* = 0.13; Fig. 1d) and significantly correlated with somatic CN alteration (SCNA) ITH (SCNA-ITH) (LUAD, *R* = 0.47, *P* = 0.039; LUSC, *R* = 0.66, *P* = 0.007) and intratumoral expression distance (ITED) (LUAD, *R* = 0.54, *P* = 0.03; LUSC, *R* = 0.59, *P* = 0.034; Fig. 1d and Methods). As both CN loss and DNA hypermethylation exhibit converging roles on gene expression, we further explored the extent and impact of these alterations during tumor evolution.

### The impact of DNA methylation on driver gene expression

**Fig. 2:**
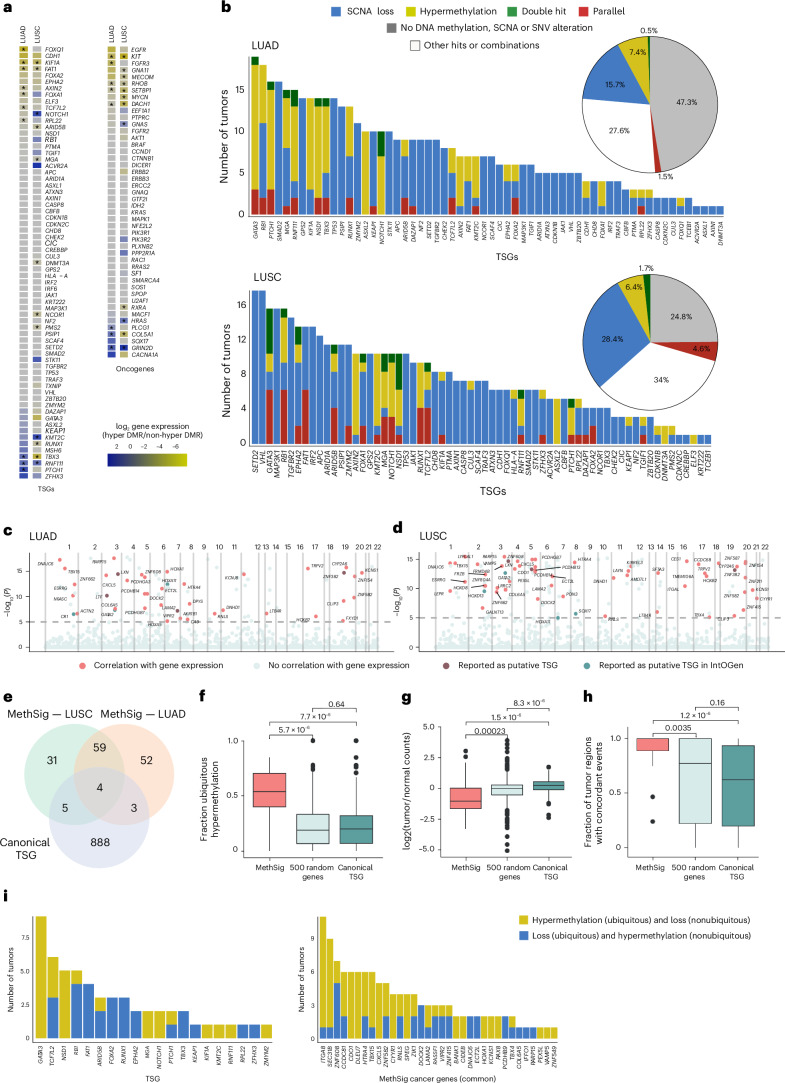
Analysis of the impact of DNA methylation on driver gene expression. **a**, Impact of promoter DMR status on gene expression for genomic TSGs (left) and oncogenes (right) for LUAD and LUSC separately. Negative values indicate decreased expression in samples where the gene promoter is hypermethylated (yellow); positive values indicate increased expression when the gene promoter is hypermethylated (blue). *P < 0.05 (*t*-test). **b**, Number of LUAD and LUSC tumors with CN loss (blue) or promoter hypermethylation (yellow) in genomic TSGs. Parallel events are defined as promoter hypermethylation and CN loss occurring in different regions of the same tumor (red). Double-hit events are defined as tumors exhibiting promoter hypermethylation and CN loss in the same region (green). Other combinations of events, including CN gains, mutations or promoter hypomethylation and combinations thereof (white), are shown. The pie chart summarizes the percentage of each type of event for all genomic TSGs. **c**,**d**, Manhattan plots illustrating the top MethSig cancer genes in LUAD (**c**) and LUSC tumors (**d**). *P* = 0.05 is indicated by the dashed horizontal line. **e**, Venn diagram showing the overlap between MethSig cancer genes and canonical genomic TSGs. **f**, Using multi-region DNA methylation data, the fraction of ubiquitous DNA hypermethylation of all MethSig cancer genes, the random set of genes and canonical TSGs, are reported (*t*-test). **g**, Relationship between the expression in tumor versus normal tissue for the MethSig cancer genes, for the random set of genes and for canonical TSGs (*t*-test). **h**, Percentage of regions exhibiting concordant alterations for both DNA hypermethylation and SCNAs in MethSig cancer genes, in the random set of genes and in canonical TSGs. Concordant events include DNA hypermethylation and CN loss, or hypomethylation with CN gain and amplification (*t*-test). The box plot shows the median, IQR (Q1–Q3), the whiskers extending to 1.5 times the IQR and outliers beyond this range. **i**, Number of tumors with ubiquitous/nonubiquitous DNA hypermethylation and CN loss events in MethSig cancer genes and canonical TSGs, used to determine the relative timing of the co-occurrence of these alterations in NSCLC.

**Extended Data Fig. 3:**
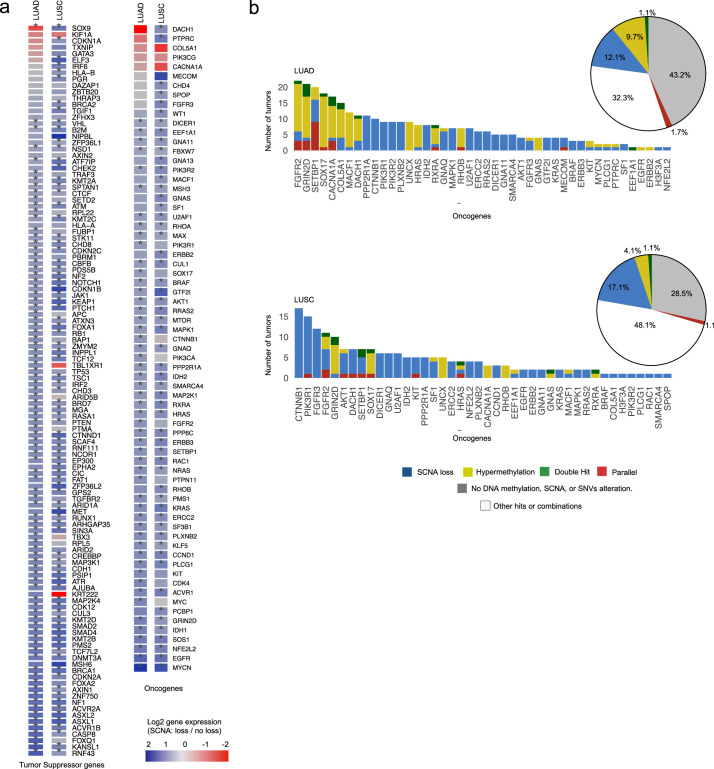
Convergent DNA methylation and genomic alterations in drivers. **a**) Impact of SCNA loss status on gene expression for genomic TSGs (left) and oncogenes (right) for LUAD and LUSC separately. Negative values indicate decreased expression in tumors where the gene is lost by SCNA, positive values indicate increased expression in tumors where the gene is lost by SCNA, *P* < 0.05 (*) (t-test). **b**) Number of tumors with alterations based on SCNA loss (blue) or promoter hypermethylation (yellow) in genomic oncogenes. Parallel events, defined as hypermethylation and copy number loss occurring in different regions of the same tumor (red). Double hit events, defined as tumors exhibiting promoter hypermethylation and somatic copy number loss in the same region (green); other combinations of events, such as somatic copy number gains, mutations or promoter hypomethylation events and combinations thereof (white). Pie chart, summarising the percentage of each event for all genomic oncogenes.

To explore the impact of DNA methylation on gene expression, we assessed differentially methylated regions (DMRs)^20^ in tumor versus NAT, identified by separately binning promoter or enhancer CpGs into neighborhoods (Methods). Unlike the significant reduction in expression of canonical NSCLC cancer genes associated with CN loss, most oncogenes and tumor suppressor genes (TSGs) did not exhibit promoter hypermethylation-dependent reductions in gene expression (Fig. 2a and Extended Data Fig. 3a), which is in line with previous reports^10^. Compared to enhancers, DNA methylation of promoters affected the expression of more TSGs (Supplementary Fig. 3). The relative infrequency of promoter hypermethylation-dependent reductions in TSG gene expression (LUAD, 7 of 68 genes; LUSC, 9 of 68 genes), together with the positive correlation between ITMD and SCNA-ITH, led us to hypothesize that a more intricate interplay may exist between SCNAs and differentially methylated promoter regions during tumor evolution.

**Extended Data Fig. 4:**
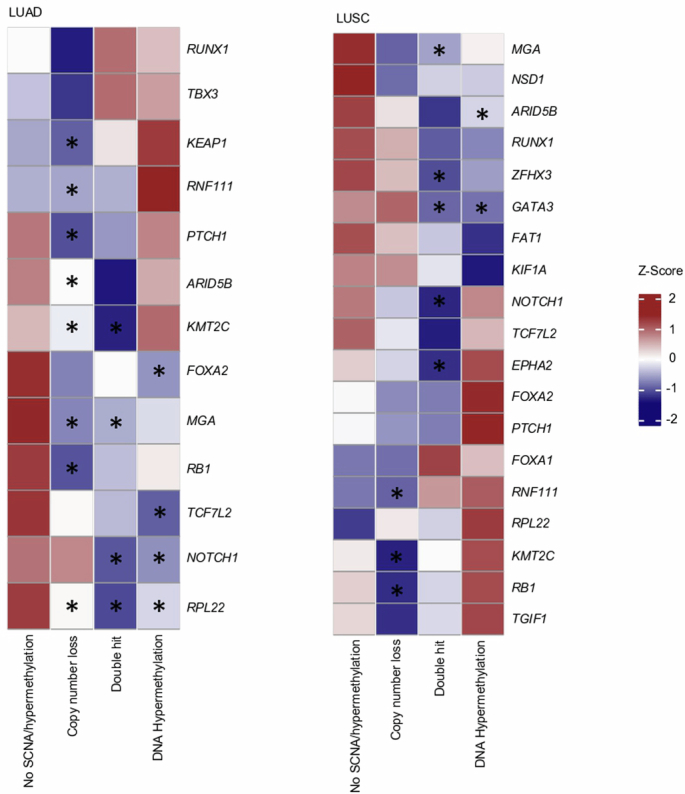
Heatmap of gene expression by copy loss and/or DNA methylation. TSG expression in samples with at least 2 tumor regions per category in LUAD (left) and LUSC (right). * indicates significance of the expression decrease relative to samples with no hypermethylation or copy number loss observed based on RRBS and WES analyses using a linear mixed model analysis. The colour scale (Z-score) is standardised by rows to allow comparisons within the same gene, with 0 being the mean value.

To study the mechanisms of convergent evolution affecting the expression of canonical TSGs, we first distinguished intratumor parallel evolution, where multiple independent mechanisms affect a locus across tumor regions, from double hits in the same tumor region. Among 68 lung cancer canonical TSGs for which we had DNA methylation and SCNA coverage, 61 of 68 were affected by either CN loss or hypermethylation in more than one tumor. Furthermore, 19 of 68 TSGs showed evidence of parallel evolution in at least two tumors. In LUSC, a greater degree of interplay between DNA hypermethylation and CN loss was evident for TSGs (6.3%) compared to oncogenes (2.2%) (*P* = 3.09 × 10^−5^; chi-squared test; Fig. 2b and Extended Data Fig. 3b). More parallel convergent events affected TSGs (for example, *FAT1*, *ZMYM2* and *EPHA2*) in LUSC (4.6%) compared to LUAD tumors (1.5%) (*P* = 5.06 × 10^−7^; chi-squared test; Fig. 2b). We next examined the impact of these concordant alterations on gene expression by applying a linear effects model to the multi-region samples. We observed a synergistic effect of CN loss and DNA hypermethylation (double hits) on the expression of *RPL22* and *MGA* in LUAD and *EPHA2* and *MGA* in LUSC (Extended Data Fig. 4). Taken together, these data suggest that in NSCLC, genomic and epigenomic mechanisms can act in parallel to abrogate TSG function.

**Extended Data Fig. 5:**
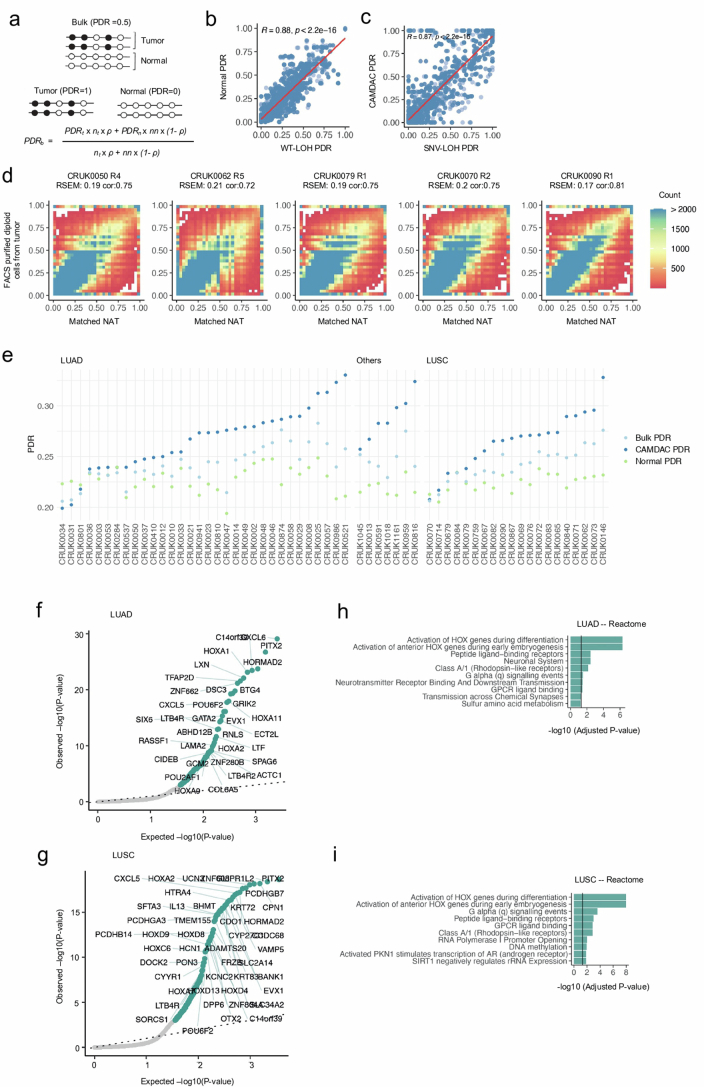
Identification of candidate DNA methylation cancer genes using MethSig. **a**) Application of CAMDAC principles to PDR. Bulk PDR (PDR_b_) can be described as a combination of the tumor PDR (PDR_t_) and normal PDR (PDR_n_) weighted by the copy number and purity. **b** and **c**) Normal and CAMDAC PDRs correlated with PDRs estimated from WT (WT-LOH PDR) and mutated reads (SNV-LOH PDR) respectively in regions with loss of heterozygosity (LOH) phased to SNVs. **d**) Correlation between PDR estimated from purified diploid cell populations from five tumor samples experimentally separated using FACS (Methods) vs. matched normal adjacent tissue (NAT). **e**) Plots showing the median PDR per tumor for bulk (PDR_b_), CAMDAC tumor (PDR_t_) and normal (PDR_n_) data. In concordance with CAMDAC principles, CAMDAC PDR (PDR_t_) levels are usually higher than the PDR_b_ when the PDR_n_ from adjacent tissue is lower than the PDR_b_. **f**) and **g**) Q-Q plot showing top significant MethSig cancer genes in LUAD and LUSC respectively. **h** and **i**) Top enriched Reactome pathways in LUAD and LUSC respectively.

As only 24.6% of established genomic TSGs were hypermethylated in more than one tumor (Fig. 2b), we next sought to identify new candidate TSGs regulated by DNA hypermethylation. Candidate DNA methylation drivers were derived using the MethSig algorithm^10^, which we built on using CAMDAC. To avoid confounded inputs, only the tumor region with the highest purity per patient was used for this analysis. Additionally, we applied CAMDAC principles to the proportion of discordant read (PDR) estimates to obtain tumor-specific signals (Methods and Extended Data Fig. 5a–e).

Using this approach, we identified 99 and 118 candidate DNA methylation cancer genes in LUAD and LUSC, respectively (Fig. 2c,d, Extended Data Fig. 5f,g and Supplementary Tables 5 and 6). Of the 63 genes identified in both subtypes, there was a significant enrichment of genomic TSGs compared to a set of random genes (*P* = 0.0422; Fisher’s exact test; Fig. 2e); 12 (including *ZNF382*, *LXN*, *RASSF1* and *CDO1*) had been previously reported as TSGs using genomic data alone. MethSig cancer genes were also enriched in developmental genes (for example, *PAX6*, *PAX8* and *TBX4*), suggesting a potential role for DNA methylation in cell plasticity (Extended Data Fig. 5h,i). LUAD MethSig cancer genes specifically exhibited a significant enrichment in *HOX* genes, which demonstrated increased methylation in samples with reduced tumor-infiltrating lymphocytes (TILs) (*P* = 0.0065; Mann–Whitney *U*-test; Supplementary Fig. 4) as reported previously^21^.

MethSig cancer genes, identified by selecting a single region per tumor, were more ubiquitously methylated within tumors compared to canonical TSGs or a selection of 500 random genes (*P* = 7.70 × 10^−6^ and 5.70 × 10^−6^, respectively; *t*-test; Fig. 2f). These data suggest that candidate methylation cancer genes are strongly selected for, or are relatively early events in tumor evolution. Additionally, MethSig cancer genes were more strongly downregulated in tumor samples compared to canonical TSGs or the random selection of genes (*P* = 1.50 × 10^−6^ and 2.30 × 10^−4^ respectively; *t*-test; Fig. 2g). We observed no differences in the calling of DMRs for MethSig cancer genes when using the isolated AT2 and BSC populations compared to bulk NAT (Supplementary Fig. 5).

We next determined the extent of interplay between DNA methylation and SCNAs affecting these candidate driver genes. Specifically, hypermethylation occurring with CN loss was defined as concordant, whereas hypermethylation occurring with CN gain was defined as discordant. MethSig cancer genes exhibited a higher proportion of concordant events than canonical TSGs or the selection of random genes (*P* = 1.2 × 10^−6^ and 3.5 × 10^−3^, respectively; *t*-test), highlighting that parallel mechanisms might affect the expression of these genes (Fig. 2h).

To compare the convergence between genomic alterations and DNA methylation events in canonical TSGs versus MethSig cancer genes, their relative timing was estimated by leveraging the multi-region nature of the sequencing data. We focused on the 38 MethSig cancer genes for which hypermethylation and CN loss each occurred in at least one tumor region. For 13 of 34 MethSig cancer genes, including *ITGA8* and *CXCL5*, we observed ubiquitous DNA hypermethylation across all regions together with nonubiquitous (that is, subclonal) CN loss (84 events of clonal hypermethylation with subclonal loss and 27 events of clonal CN loss with subclonal hypermethylation), whereas 8 of 20 canonical TSGs, including *FAT1*, exhibited ubiquitous CN loss with subclonal hypermethylation (28 events of clonal hypermethylation with subclonal loss and 38 events of clonal CN loss with subclonal hypermethylation). These data suggest that like the clonal disruption of canonical TSGs, hypermethylation of MethSig cancer genes may be early events in NSCLC, often preceding subclonal CN loss of the same gene (*P* = 2.84 × 10^−2^; chi-squared test; Fig. 2i).

### Divergence of DNA methylation and CN alterations

**Extended Data Fig. 6:**
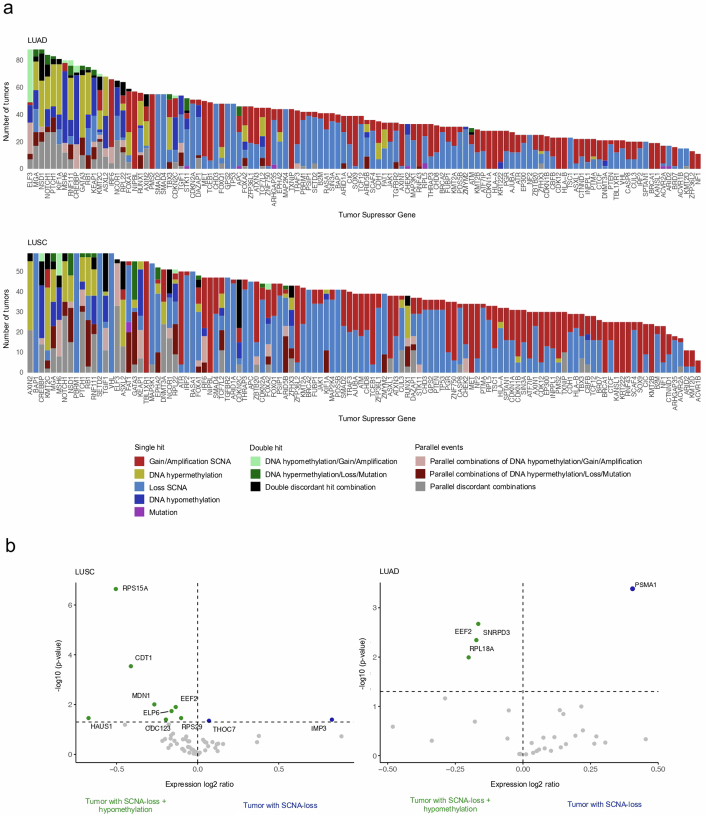
Divergent interplay between DNA methylation status and genomic alterations in genomic driver genes. **a**) Number of concordant and discordant combinations of copy number, DNA methylation, and inactivating mutations impacting canonical TSGs in LUAD and LUSC. Double hits are defined as the combination of more than two types of concordant events identified within the same tumor region. Parallel events refer to concordant events identified in different regions of the same tumor. **b**) Differential expression analysis of essential genes comparing tumor regions with both hypomethylated DMRs and SCNA loss versus tumor regions with SCNA loss alone in LUAD and LUSC (t-test).

The limited concordance between DNA methylation and genomic events at canonical TSGs (Fig. 2h) prompted us to explore the prevalence of discordant mechanisms of interplay between these alterations. Co-occurring CN loss and hypomethylation events were more prevalent in LUSC, affecting TSGs including *NCOR1* (29 of 59 tumor regions), *CDKN2C* (28 of 59 tumor regions), *CREBBP* (26 of 59 tumor regions) and *RPL22* (9 of 59 tumor regions) (Extended Data Fig. 6a). Interestingly, *RPL22* (1p36.3), *NCOR1* (17p12) and *CDKN2C* (1p32.3) are located in proximity to known aphidicolin-induced common fragile sites, such as FRA1A, FRA17 and FRA1B, respectively^22,23^. We also observed an enrichment of essential genes such as *RPS15A*, *CDT1* and *MDN1* to be under DNA hypomethylation-dependent dosage compensation in regions of CN loss in LUSC (Extended Data Fig. 6b).

**Fig. 3:**
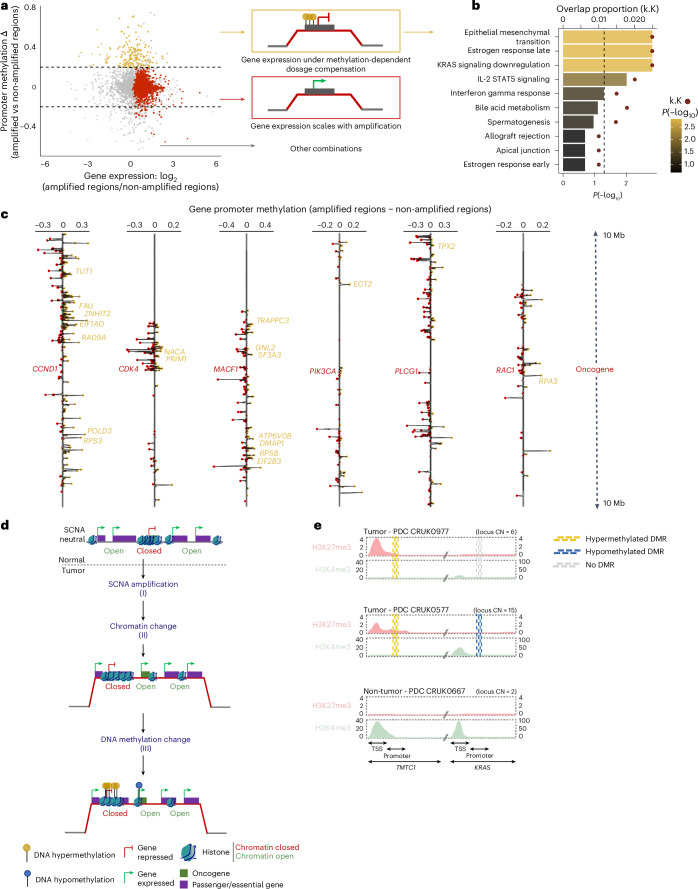
Divergent interplay between DNA methylation and CN alterations. **a**, Difference in median promoter methylation for genes when amplified versus when not amplified (*y* axis). A value greater than 0.2 indicates increased DNA methylation when amplified. The *x* axis indicates the ratio of gene expression between amplified versus non-amplified regions. Positive values indicate gene expression scales with CN amplification. Genes highlighted yellow are potentially under DNA-methylation-dependent dosage compensation, as their methylation, but not their expression, scales with CN. Genes with expression levels that scale with CN but do not scale with DNA methylation are highlighted red. **b**, Hallmarks in cancer functional enrichment of genes potentially under DNA-methylation-dependent dosage compensation. The bar lengths represent the *P* value; the proportion of overlap between the subset of genes (*k*) and the gene sets defining the hallmarks (*K*) are indicated by a red dot. **c**, Gene promoter methylation difference between samples with and without amplification located within 20 Mb of amplified oncogenes with expression levels that scale with CN, which are labeled red (HUGO Gene Nomenclature Committee name). Essential genes extracted from the Achilles project dataset are labeled yellow (HUGO Gene Nomenclature Committee name). **d**, Schematic illustrating the potential cooperation between CN alterations and DNA methylation around oncogenes. CN changes at the oncogene locus could trigger a focal AllChAT, affecting co-amplified essential and passenger genes. **e**, Validation of AllChAT on the gene pair *TMTC1* as a passenger of the amplified oncogene *KRAS*, in primary cell cultures derived from patient tumors CRUK0977 and CRUK0577, and from a non-tumor-tissue-derived primary cell culture from patient CRUK0667. The CN for each locus is indicated numerically. The repressive histone mark H3K27me3 to identify closed chromatin (red), and the active histone mark H3K4me3 to identify open chromatin H3K4me3 (green), were extracted from the Integrative Genomics Viewer and illustrated using BioRender. The intensity of both histone marks was normalized according to the CN. Assessment of DNA methylation status in the promoter region of each gene was performed using the non-tumor PDC as a control for the two tumor PDCs.

**Extended Data Fig. 7:**
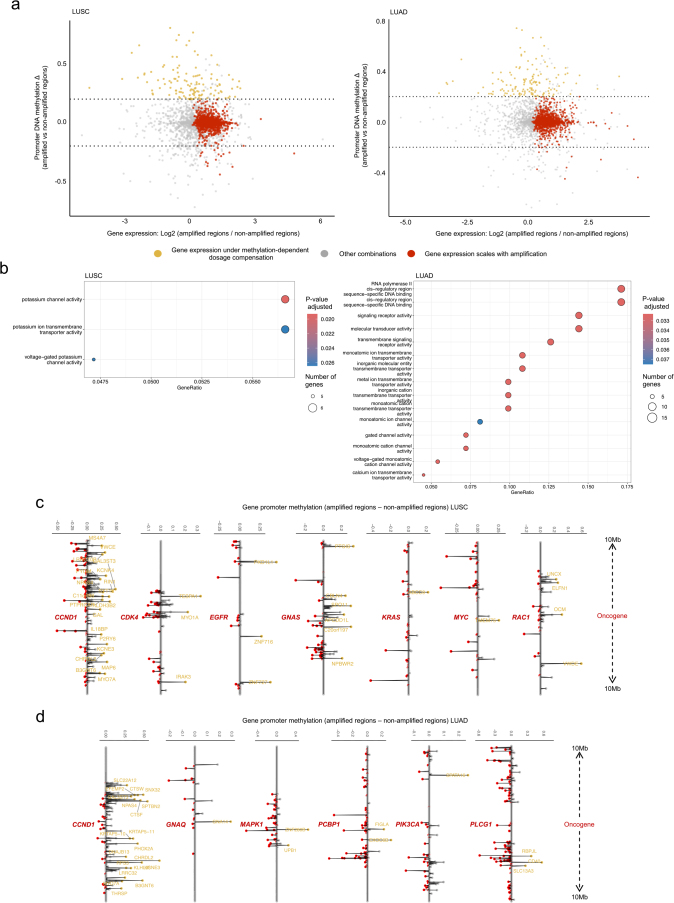
Divergent interplay between DNA methylation and copy number in amplified regions in LUAD and LUSC separately. **a**) Difference in median promoter DNA methylation (*y* axis) versus log2-fold change in median expression for genes when amplified versus when not amplified (*x* axis). Genes highlighted in yellow are potentially under DNA methylation-dependent dosage compensation. Genes with expression levels that scale with copy number and do not scale with DNA hypermethylation are highlighted in red; LUSC (left); LUAD (right). **b**) GO terms highlighting the enriched pathways for genes under DNA methylation-dependent dosage compensation in LUSC (left) and in LUAD (right). **c, d**) DNA methylation-associated dosage compensation of genes co-amplified within 20 Mb of oncogenes in **c**) LUSC and **d**) LUAD. Genes with a DNA methylation difference > 0.2 when amplified versus non-amplified are labelled in yellow. Genes with expression levels that scale with copy number and do not scale with DNA hypermethylation are highlighted in red.

We next explored the interplay between DNA methylation and gene expression at loci that are amplified (Methods). Genes with higher expression levels and no increase in DNA methylation when amplified were enriched in oncogenes (Fig. 3a, red dots). Genes with reduced or equal expression, but with increased DNA methylation when amplified may be subject to DNA methylation-dependent dosage compensation (Fig. 3a, yellow dots). Gene set enrichment analysis revealed that these dosage-compensated yellow genes were enriched in pathways related to epithelial–mesenchymal transition, KRAS signaling, immune pathways (Fig. 3b) and several transmembrane channels in both LUSC and LUAD (Extended Data Fig. 7a,b).

Focusing on regions with recurrently amplified oncogenes (Fig. 3c), we hypothesized that DNA hypermethylation could be part of a mechanism to maintain neighboring co-amplified, but dosage-sensitive, genes near their basal expression level. We calculated the average difference in DNA methylation rates at frequently amplified regions between tumor regions with and without the amplification (Methods). Oncogenes with expression scaling with amplification, such as *RAC1* and *CDK4*, were less methylated when amplified (*P* = 1.53 × 10^−5^ and 5.63 × 10^−4^, respectively; Mann–Whitney *U*-test) compared to non-amplified tumor regions. We identified oncogene-proximal genes under dosage compensation by DNA methylation associated with amplification of *CCND1* in both LUSC and LUAD and *CDK4*, *KRAS*, and *GNAS* exclusively in LUAD (Extended Data Fig. 7c,d). Dosage-compensated essential genes, such as *RPS3*, located in oncogene-proximal regions (for example, *CCND1*; Fig. 3c), were significantly enriched compared to other genomic regions (*P* = 0.028; chi-squared test). These data suggest a potential interplay between CN alterations and DNA methylation, whereby changes in CN at the oncogene locus could trigger a phenomenon that we have called an allosteric chromatin activity transition (AllChAT) affecting the DNA methylation status of neighboring passengers genes (Fig. 3d).

To investigate AllChAT, we performed chromatin immunoprecipitation followed by sequencing (ChIP–seq) for H3K27me3 to identify closed chromatin regions, and H3K4me3 for open chromatin in tumor patient-derived cells (PDCs) from TRACERx tumors (CRUK0977 and CRUK0557), and a PDC from NAT (CRUK0667). Oncogenes such as *CDKN1B*, *FGFR1* and *JAK2* were co-amplified and associated with chromatin opening and hypomethylation when the locus was amplified compared to when it was not. Additionally, we found that essential passenger genes, including *NOP2*, *DCTN6* and *FOXD4*, were associated with closed chromatin and increased DNA methylation at the same respective loci when amplified compared to when not amplified in tumor PDCs compared to normal PDCs (Supplementary Table 7). We also observed AllChAT at the locus of the MethSig cancer gene *TMTC1* after co-amplification with the *KRAS* oncogene in both tumor PDCs, along with concomitant changes in promoter methylation (Fig. 3e). In TRACERx tumor tissues (Fig. 3c), we observed evidence for methylation-dependent dosage compensation in *LRRC34* when co-amplified with the *PI3KCA* oncogene in both tumor PDCs (Supplementary Table 7). Finally, using 137 samples from five tissue types from the EpiATLAS public dataset^24^, we observed co-amplification of the essential gene *SMC4* with the oncogene *MECOM*, associated with recruitment of the closed H3K27me3 mark around *SMC4* and its concomitant hypermethylation. Taken together, these data further support a role for AllChAT in the regulation of essential genes during tumor evolution.

### M_R_/M_N_ stratifies genes under selection by DNA methylation

**Fig. 4:**
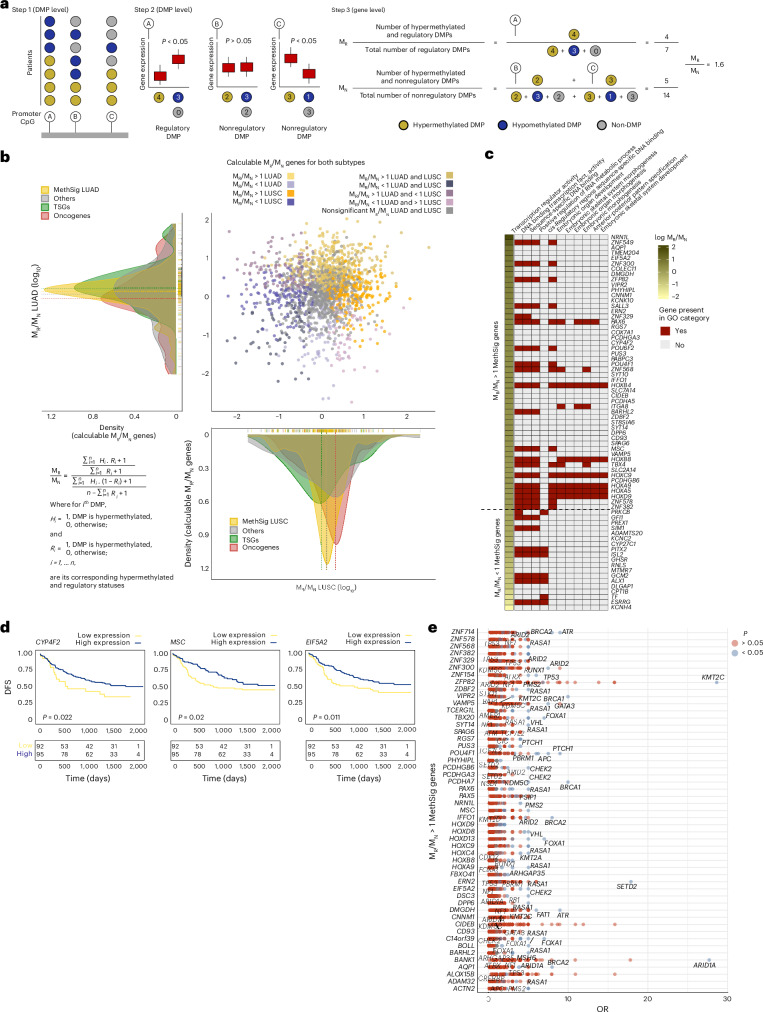
Identification of cancer-related disruption events by applying M_R_/M_N_ to MethSig. **a**, Schematic of the development of the M_R_/M_N_ metric. (1) The DMP status is assigned for each CpG in the gene promoter across the cohort. (2) Each DMP is characterized as regulatory or nonregulatory based on whether hypermethylation of the CpG reduces gene expression of the cognate gene across the cohort. (3) M_R_ and M_N_ values for each gene are calculated based on the aggregated DNA methylation status of regulatory and nonregulatory CpGs in each gene promoter across the entire cohort. **b**, log–log scatter plot displaying the common calculable M_R_/M_N_ ratios for each gene in LUAD (*y* axis) and LUSC (*x* axis). On the density plot, subtype-specific calculable M_R_/M_N_ ratios according to genes are indicated. The formula for determining the M_R_/M_N_ ratio for each gene is illustrated in the lower left corner. The colors in the log–log scatter plot represent the direction of deviation of M_R_/M_N_ from 1 for each subtype and its significance. **c**, Functional enrichment analysis with Gene Ontology (GO) terms for MethSig genes with M_R_/M_N_ > 1 (top) and M_R_/M_N_ < 1 (bottom). **d**, Kaplan–Meier curves based on the expression of the MethSig cancer genes with an M_R_/M_N_ > 1 (*CYP4F2*, *MSC* and *EIF5A2*) associated with worse DFS in the TRACERx cohort (multivariate Cox analysis). **e**, Odds ratio (OR) highlighting the co-occurrence of promoter DNA hypermethylation events for M_R_/M_N_ > 1 MethSig cancer genes and driver mutations in canonical TSGs in LUAD. Significant co-occurrences are labeled.

The enrichment of hypermethylated essential genes neighboring oncogenes that scale with amplification prompted us to closely evaluate the impact of DNA hypermethylation on gene expression. We derived a new metric to identify genes subject to cancer-associated disruption of gene expression. M_R_/M_N_ assigns genes by determining the ratio of regulatory hypermethylated DMPs over nonregulatory hypermethylated DMPs located in gene promoter regions (Fig. 4a, Supplementary Table 8 and Methods), analogous to dN/dS in protein-coding genes. For most genes, M_R_/M_N_ is approximately 1 (Fig. 4b and Supplementary Table 8). Like dN/dS, we hypothesize that this ratio may provide insights into the nature and direction of selection. Specifically, an M_R_/M_N_ ratio greater than 1 (false discovery rate (FDR) < 0.05) suggests preferential hypermethylation of regulatory DMPs, while M_R_/M_N_ ratios smaller than 1 (FDR < 0.05) suggest enrichment of hypermethylation at nonregulatory DMPs that do not affect expression.

**Extended Data Fig. 8:**
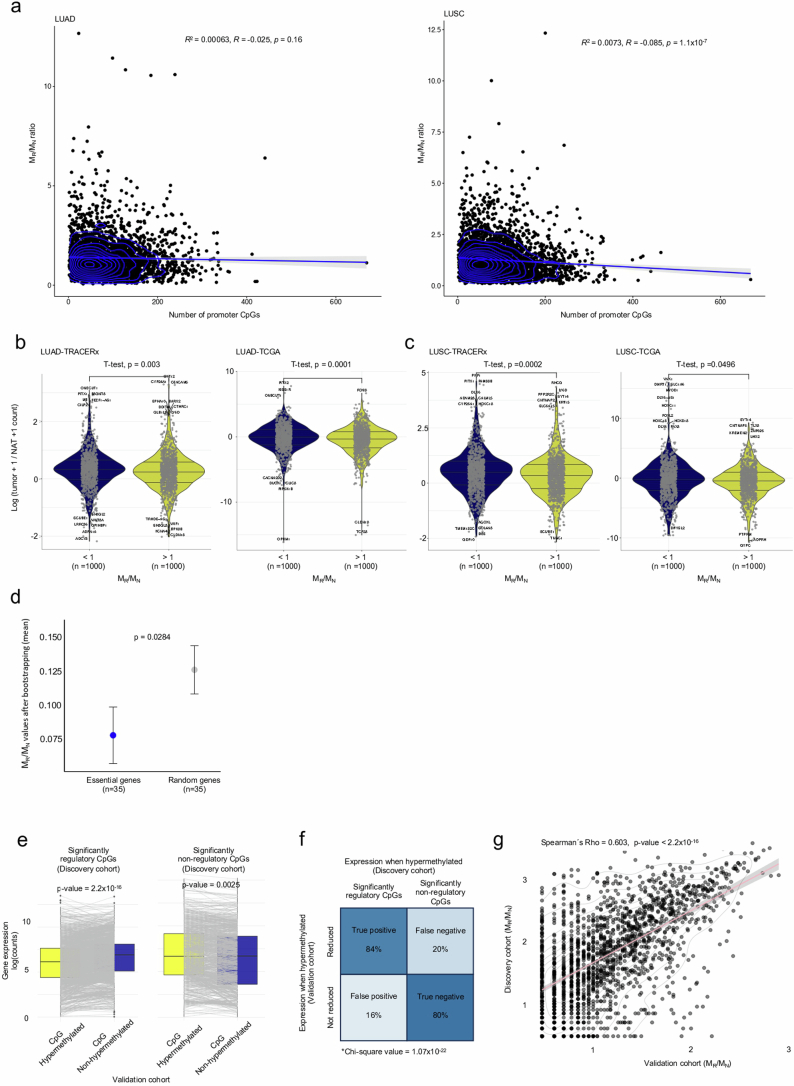
Implementation of M_R_/M_N_ to stratify genes under DNA methylation-dependent regulatory selection. **a**) Linear regression between the logarithm of the number of promoter CpGs and the M_R_/M_N_ ratio per gene in LUAD and LUSC (95% confidence intervals are indicated in grey). **b**) Gene expression ratio between the tumor and the normal adjacent tissue (NAT) for the top 1000 genes with highest M_R_/M_N_ and bottom 1000 M_R_/M_N_ in the LUAD TRACERx RRBS cohort and the LUAD TCGA cohort. **c**) Gene expression ratio between the tumor and the NAT for the top 1000 genes with highest M_R_/M_N_ and bottom 1000 M_R_/M_N_ in the LUSC TRACERx RRBS cohort and the LUSC TCGA cohort (t-test). **d**) Mean value ± SEM of M_R_/M_N_ of known essential genes extracted from the Achilles dataset project versus the mean value ± SEM of M_R_/M_N_ from a random iteration of selected genes (t-test). **e**) Validation of the promoter CpG assignments (regulatory and non-regulatory) using an additional 17 regions from 10 LUAD from the TRACERx cohort as an independent validation cohort using CpGs significantly assigned as regulatory (left boxplot), and significantly non-regulatory (right boxplot) in the discovery cohort (t-test). **f**) Confusion matrix showing the percentages of CpGs selected in panel ‘e’ in both the discovery and validation cohorts that are associated with reduced gene expression (or not) when hypermethylated versus when non hypermethylated. For the validation cohort, ‘reduced’ CpGs have been assigned when the expression ratio between when the CpG is hypermethylated versus when it is not is less than 0.5, while ‘Not reduced’ has been considered when the ratio is greater than 1.5. Significance has been evaluated using a chi-squared test. **g**) Validation of the M_R_/M_N_ metric by comparing the value of M_R_/M_N_ in the discovery vs the validation cohort (Correlation coefficient calculated using the Spearman method, 95% confidence intervals are indicated in grey).

We observed no relationship between the number of CpGs studied and the M_R_/M_N_ ratio, ensuring that the M_R_/M_N_ metric is robust to promoter CpG content (Pearson’s *R* = −0.025 for LUAD and *R* = −0.085 for LUSC; Extended Data Fig. 8a). We next compared the expression level of genes with M_R_/M_N_ ratios greater than 1 versus those with ratios smaller than 1 in tumors compared to matched NATs. As expected, genes with an M_R_/M_N_ greater than 1 exhibited a significantly stronger downregulation of expression in the tumor compared to genes with an M_R_/M_N_ smaller than 1, observed in both LUAD and LUSC (*P* = 3.0 × 10^−3^ and *P* = 2.0 × 10^−4^, respectively; Extended Data Fig. 8b,c). Importantly, this effect was consistently observed in the LUAD and LUSC datasets from The Cancer Genome Atlas (TCGA) (*P* = 1.0 × 10^−4^ and *P* = 4.9 × 10^−2^, respectively; Extended Data Fig. 8b,c). Consistently, essential genes exhibited significantly lower M_R_/M_N_ values compared to a random set of genes (*t*-test, *P* = 0.028; Extended Data Fig. 8d), suggesting selection against DNA methylation-dependent reduced expression for essential genes during tumor evolution.

To validate the M_R_/M_N_ metric, we performed RNA-sequencing (RNA-seq) and RRBS on an independent cohort of 17 TRACERx LUAD samples from ten patients and matched NATs. DMP assignments in the test cohort were maintained in the validation cohort (differential expression in hypermethylated versus non-hypermethylated samples in a paired *t*-test; *P* < 2.2 × 10^−16^ for regulatory DMPs; Extended Data Fig. 8e) with a true positive rate of 84%, a true negative rate of 80%, a specificity of 83.3% and sensitivity of 80.7% (chi-squared *P* < 1.07 × 10^−22^; Extended Data Fig. 8f). Furthermore, we observed a significant correlation between the M_R_/M_N_ ratio for each gene between the test and validation cohorts (Spearman’s rho = 0.603, *P* < 2.2 × 10^−16^; Extended Data Fig. 8g).

### Cancer-related MethSig genes disrupted by DNA methylation

MethSig cancer genes demonstrated a broad range of M_R_/M_N_ ratios in LUAD and LUSC (Fig. 4b, yellow). We hypothesize that, of these candidate methylation drivers, those with a strong correlation between epimutations and gene expression are more likely to be under positive selection. Furthermore, despite exhibiting M_R_/M_N_ ratios smaller or greater than 1, several MethSig cancer genes were alternatively under positive selection for deleterious variants, as defined by their higher dN/dS ratios (Supplementary Fig. 6a).

We evaluated whether applying M_R_/M_N_ to MethSig cancer genes could further stratify this functionally diverse pool of DNA methylation cancer genes. In LUAD, MethSig cancer genes with an M_R_/M_N_ greater than 1, including the *HOX* genes *PAX6* and *ITGA8*, were enriched for cancer progression pathways, such as motility, tissue development and morphogenesis, and transcription regulation. On the other hand, MethSig cancer genes with an M_R_/M_N_ smaller than 1 revealed enrichment of only transcriptional regulatory genes and were enriched at amplified loci (Fig. 4c and Supplementary Fig. 6b,c). In the bulk analyses, genes with an M_R_/M_N_ greater than 1 were enriched in stromal signatures; however, this enrichment was no longer observed after CAMDAC-based purification (Supplementary Fig. 6d). Leveraging a small interfering RNA viability screen in the LUAD PC9 cell line^25^, we observed that depletion of the MethSig cancer genes *ITAG8* and *SLC7A15* with an M_R_/M_N_ greater than 1 exhibited the highest proliferation rates among all MethSig LUAD genes.

To further investigate the impact of MethSig cancer genes with an M_R_/M_N_ greater than 1, we leveraged methylation values and their associated gene expression levels in the TRACERx RRBS cohort to dichotomize the larger TRACERx RNA-seq cohort (Methods). This approach allowed us to assess whether stratifying MethSig cancer genes according to M_R_/M_N_ status could reveal differences in disease-free survival (DFS). Unlike the MethSig cancer genes with an M_R_/M_N_ smaller than 1, three of the 52 MethSig cancer genes with an M_R_/M_N_ greater than 1 (*CYP4F2*, *MSC* and *EIF5A2*) were associated with worse survival in a multivariate Cox analysis (*P* = 0.022 for *CYP4F2*, *P* = 0.02 for *MSC* and *P* = 0.011 for *EIF5A2;* Fig. 4d and Supplementary Fig. 6e).

Finally, we assessed which genomic alterations in TRACERx tumors co-occurred with these candidate DNA methylation driver events. In LUAD, driver mutations in *STK11* and *KDM5C* resided in tumor regions with predicted hypermethylation of *ZNF714*, *MSC* and *EIF5A2* MethSig cancer genes with an M_R_/M_N_ greater than 1 (Fig. 4e). In LUSC, a predominance of tumor regions with driver mutations in *ATR* and *KMT2D* was observed along with predicted hypermethylation of *PITX2* or *VIRP2*, MethSig cancer genes with an M_R_/M_N_ greater than 1 (Supplementary Fig. 7). Expansion of our dataset to a cohort of preinvasive lesions^9^ revealed that although the *VIPR2* and *ZNF714* genes were already methylated in preinvasive lesions, co-occurrence with driver mutations in *CDKN2A* and *STK11*, respectively, was only evident in LUAD (*P* = 2.5 × 10^−03^ and *P* = 1.9 × 10^−07^; Fisher’s exact test; Fig. 4e). Notably, these mutations were relatively infrequent in the preinvasive cohort. These results suggest that methylation of these genes with an M_R_/M_N_ greater than 1 may occur early in tumorigenesis and could enable prediction of subsequent genomic trajectories.

## Discussion

To capture the complex interplay between the genome and epigenome in NSCLC, we leveraged the high sequencing depth provided by RRBS on 217 tumor samples from 59 lung TRACERx patients and applied the CAMDAC deconvolution tool^17^ to enrich cancer-cell-specific methylomes. Unsupervised hierarchical clustering of the most variable CpGs sites revealed a clear separation between histological subtypes, highlighting the benefit of the tumor deconvolution strategy.

We developed ITMD, an approach to evaluate the degree of intratumoral DNA methylation heterogeneity. CAMDAC ITMD scores were not affected by sampling bias, sequencing coverage, CN or tumor purity, probably because they are not dependent on methylation signals from different cell types within the tumor, unlike other approaches^26,27^. Second, while other ITH studies relied on entropy^26–28^, we observed heterogeneity of CpG sites in multiple regions of the same tumor. Finally, unlike similar ITH scores that use SNVs and CNs for functional validation^29^, we additionally encompassed the impact of methylation heterogeneity on the heterogeneity of global gene expression.

Through integrating DNA methylation and CN data, we identified several canonical TSGs, such as *STK11* and *CDKN1B*, which were most often targeted by a single alteration, in line with previous reports of their haploinsufficiency^30,31^.

Using CAMDAC cancer-cell-specific methylomes as input for MethSig, we observed significant enrichment of hypermethylated candidate NSCLC cancer genes known to encode differentiation and developmental transcription factors, such as *PCDHGA3* and *EVX1*, and in *ZNF-154*, which may affect plasticity^32^. These MethSig events probably reflect histology-specific early DNA methylation events. Early inactivation of developmental genes may facilitate transformation through mechanisms such as preventing or reverting lineage differentiation and locking cells into a perpetuated stem-cell-like state, increasing their propensity to become transformed by additional oncogenic events^33^. Our findings further emphasize the potential of incorporating epigenetic modulators into combination therapy.

To assess the extent of positive selection of DNA hypermethylation at gene expression regulatory versus nonregulatory CpGs in gene promoters, we developed M_R_/M_N_, a metric that relies on the expectation that expression-associated DMPs are more likely to be under positive selection. We hypothesize that genes with an M_R_/M_N_ greater than 1 may confer a selective advantage.

To date, dosage compensation studies have primarily focused on epigenetic regulation of the X chromosome, such as methylation-dependent dosage compensation and downregulation of *SOX1*, which is known to influence patient prognosis in breast cancer^34,35^. Dosage compensation by hypermethylation of genes amplified by virtue of their location proximal to an oncogene was enriched in essential genes. Many of these essential genes encode proteins that are part of complexes and are probably under selective pressure to maintain complex stoichiometry. This potential cooperation between genetic and epigenetic events may parallel the concept of allostery, which was introduced over a century ago to describe the phenomenon whereby one molecule affects the binding affinity of another molecule to a protein^18^. The process involves one or more cooperative changes at sites that are spatially separated from the target site, triggering an allosteric activity transition in the molecule. Extending the same concept to chromatin, we hypothesize that cooperation between local changes in CN and DNA methylation around one gene can trigger an *in*-*cis* focal AllChAT affecting a nearby gene, as exemplified by the essential gene *DDX42*, located 9 Mb from the oncogene *SOX9* and the *TMTC1* gene with an M_R_/M_N_ smaller than 1 located 4 Mb upstream of *KRAS*.

Our study is not without limitations. The M_R_/M_N_ metric assumes that hypermethylated DMPs associated with reduced expression are regulatory, disregarding other confounding factors, such as the impact of SNVs and CN loss. M_R_/M_N_ is also restricted to regulatory CpGs proximal to the transcription start site (TSS) regions and neglects other potential regulatory sites. In addition, 1.3% of promoter CpG sites, particularly associated with chromatin modifiers, exhibit a strong positive correlation between DNA methylation and gene expression^36^, which is not considered in our methods. Despite not observing a general correlation between the dN/dS and the M_R_/M_N_ ratio within canonical TSGs, *cis*-regulatory mutations in these promoter regions may be associated with changes in DNA methylation at the same site, which could also interfere with our metric. Despite these assumptions, our data suggest that an early DNA methylation event may commit the primary tumor to particular genomic trajectories, as suggested for *MGMT* hypermethylation preceding *KRAS* activating mutations in colorectal cancer^37^. Furthermore, the incorporation of epigenetic modifications into cancer evolution trajectories may improve our understanding of the intricate relationship between genetic and epigenetic alterations and facilitate stratification of patients with NSCLC for appropriate therapeutic regimens.

## Methods

### Patient selection for RRBS

The TRACERx study (ClinicalTrials.gov identifier: NCT01888601) is a prospective observational cohort study that aims to transform our understanding of NSCLC. It was approved by an independent research ethics committee, the National Research Ethics Service Committee London–Camden and Islington, with sponsor’s approval of the study by University College London (UCL) (research ethics committee reference no. 13/LO/1546, protocol no. UCL/12/0279, Integrated Research Approval System project ID: 138871). The design has been approved by an independent research ethics committee (no. 13/LO/1546). Written informed consent for entry into the TRACERx study was mandatory and obtained from every patient. All patients were assigned a study ID number known to the patient. We performed RRBS on 217 tumor regions from 59 patients (32 with LUAD, 20 with LUSC and seven with other NSCLC subtypes) all with matched NATs. Among the 59 patients, 31 were stage I, 14 stage II and 14 stage III. Forty-seven were ex-smokers, six current smokers and six never-smokers (Supplementary Fig. 1b). RNA-seq data were leveraged from 43 patients (129 regions) and WES data from 45 patients (159 regions) from the TRACERx cohort (Supplementary Fig. 1a).

### Dual DNA/RNA extraction

Sequential extraction of DNA and RNA was performed from the same sample using the AllPrep DNA/RNA Mini Kit (QIAGEN). Briefly, frozen samples were transferred onto cold Petri dishes on dry ice and were dissected into 20–30 mg pieces. Immediately before extraction, the freshly dissected tissue was transferred directly into homogenization tubes containing RLT plus lysis buffer. Tissue homogenization was carried out using a TissueRuptor II probe or using a bead method and by passing the lysate through a QIAshredder column (QIAGEN). The DNA extracted was eluted with 200 µl of buffer EB (no EDTA) and RNA was eluted with 200 µl of nuclease-free water and stored immediately at −80 °C. The DNA and RNA samples were quantified using a Qubit 3.0 Fluorometer (Thermo Fisher Scientific) and TapeStation system (Agilent Technologies), respectively. The integrity of DNA/RNA was assessed using the TapeStation system.

### RRBS

DNA methylation profiles were obtained using RRBS^38^ with the NuGEN Ovation RRBS Methyl-Seq System, which incorporates unique molecular identifiers facilitating single-molecule analysis and precise methylation estimates^16^. The choice of the method for DNA methylation analysis of the TRACERx cohort was driven by (1) the available sample quantity, (2) cost-efficiency, accuracy, reproducibility and feature coverage of the available methods^16^ and (3) the required depth of coverage. An inherent limitation of the targeted over-whole-methylome approaches is the reduced coverage of the non-CpG-rich regulatory regions (for example ~25% of FANTOM5 enhancers for RRBS); however, considering the trade-offs and sample and coverage constraints, RRBS was selected as the method of choice. Additionally, RRBS covers 90.02% of promoters with CpGs, making it the optimal method for studying the impact of DNA methylation on the regulation of protein-coding genes.

RRBS sequencing libraries were created by enzymatically digesting 100 ng of genomic DNA using MspI, which recognizes 5′-CCGG-3′ sequences and cleaves phosphodiester bonds upstream of CpG dinucleotides, leaving a 2-bp overhang suitable for adapter ligation. Bisulfite conversion was performed using the QIAGEN’s EpiTect Fast DNA Bisulfite Kit. Agencourt RNAClean XP magnetic beads were used to purify the converted libraries amplified using PCR. Purified libraries were quantified using the Qubit dsDNA HS Assay Kit (Invitrogen) and quality was evaluated using the Agilent Bioanalyzer High Sensitivity DNA Assay (Agilent Technologies).

FastQC v.0.11.2 (Babraham Institute, https://www.babraham.ac.uk/) was used for quality control. Adapter sequences and diversity bases were trimmed using TrimGalore v.0.6.6 and the NuGEN’s trimRRBSdiversityAdaptCustomers.py custom script (https://github.com/nugentechnologies/NuMetRRBS). Reads were aligned to the UCSC hg19 reference assembly using Bismark v.0.23.0 and Bowtie v.2-2.4.2 (refs. ^39,40^); deduplication was carried out using NuDup (https://github.com/nugentechnologies/nudup). A Nextflow pipeline to perform the alignment and quality control is available at https://github.com/ccastignani/RRBS_DNAmethylation_pipeline.

### CN-aware methylation deconvolution of cancers

The CAMDAC method^17^ was used to obtain cancer-cell-specific methylation rates from bulk RRBS data evaluating 1.8 M CpGs covered in every sample in the cohort. Absence of tumor infiltration from matched NATs was assessed using pathology and transcriptomic analyses and was used as the normal infiltrate contaminant component in the tumor.

CAMDAC deconvolution relies on ASCAT.m, a module that infers allele-specific CN from RRBS data leveraging the same principles presented in ref. ^41^. To improve ASCAT.m CN calling, we performed multi-sample phasing. In segments with an allelic imbalance in at least one sample, haplotyping was performed by taking the B allele frequency of heterozygous single-nucleotide polymorphisms. After multi-region phasing, ASCAT.m solutions for 67 samples were refitted manually and 26 samples were excluded because of low quality (low coverage or low proportion of tumor cells).

At loci with allele-specific methylation, a copy gain or loss can simultaneously result in an apparent hypomethylation or hypermethylation event, depending on whether the methylated or unmethylated copy is involved. As these allele specifically methylated loci represent 5% or less of loci and CN events at these regions may have biological meaning^17^, we included them in the concordant or discordant counts accordingly.

### Tumor-normal differential methylation analysis

Tumor-normal DMPs were identified based on a statistical test described in ref. ^17^. The CAMDAC cancer-cell-specific methylation rate (m_t_) and the adjacent normal methylation rate as proxy for the cell of origin (m_n_) were used. Significant DMPs were identified using a *P* < 0.01 and a difference threshold of 0.2 between methylation rates (that is, m_t_ − m_n_ > 0.2). DMRs were called by binning CpGs into neighborhoods and identifying DMP hotspots in these clusters. CpGs that fell within 100 bp of one another were grouped together. For each bin, the number of consecutive DMPs with an effect size above 0.2 and *P* < 0.01 were computed. Genomic bins with four or more consecutive DMPs and at least five DMPs in total were deemed DMRs. Methylation status in gene promoters (defined as starting 2.5 kb upstream and ending 250 bp downstream of the TSS) was used to compute the methylation status per gene.

### Hierarchical clustering

Unsupervised hierarchical clustering of the top 5,000 most variable CpGs, based on the s.d., was performed using the Ward’s minimum variance clustering method implemented in the R package ComplexHeatmap^42^. Bootstrap hierarchical clustering was performed using the R package pvclust (https://github.com/shimo-lab/pvclust) with the hierarchical clustering method set to ‘average’ and using a Pearson distance matrix^43^. For each analysis, we ran 1,000 bootstrap iterations and significant clusters were taken using alpha > 0.95. Cluster stability values were estimated using the clusterboot() function from the fpc R package (https://cran.r-project.org/web/packages/fpc/index.html). The use of 5,000 most variable CpGs in this analysis was representative of the variation in the cohort. Cluster stability was evaluated using the Fowlkes–Mallows index, which is used to determine the similarity between two sets of hierarchical clustering. Clusters taken from the 5,000 most variable CpGs were compared against the clusters derived from the 10,000, 20,000 and 50,000 most variable CpGs. The Fowlkes–Mallows indices of 0.97 for 5,000 versus 10,000, 0.96 for 5,000 versus 20,000, and 0.95 for 5,000 versus 50,000 were obtained.

### Intratumor heterogeneity metrics

ITED^3^ was calculated as the mean normalized gene expression correlation distance for a given tumor region paired with every other region from the same tumor^3^. Mutational and CN heterogeneity were calculated based on recently established metrics^2^. ITMDs were computed based on the pairwise Pearson distance between all CpGs across all sampled regions per tumor.

### Isolation of basal and AT2 cells from normal human tissue

Human cells derived from lobectomy tissue (TRACERx patients CRUK1231, CRUK1266, CRUK1262, CRUK1320 and CRUK1319) were isolated as described previously^44^ and either used immediately or cryopreserved before flow cytometry sorting. Cryopreserved samples underwent a 1.5-h incubation at 37 °C before staining with antibodies. Cells were blocked with anti-Fc block (Fc1, BD Biosciences) and stained with the following antibodies using a standard concentration of 0.25 µg 10^−^^6^ cells: CD45-PE (HI30, BD Biosciences); CD235a-PE (clone HIR2, BD Biosciences); CD140b-PE (clone 28D4, BD Biosciences); CD31-PE (clone WM59, BD Biosciences); EpCAM-FITC (clone VU-1D9, STEMCELL Technologies); podoplanin-APC-Cy7 (clone NC-08, BioLegend); CD166-APC (clone eBioALC48, Thermo Fisher Scientific); CD49f-PE-Cy7 (clone GoH3, Thermo Fisher Scientific); and propidium iodide (BD Biosciences). Samples were sorted on FACSAria cell sorters (BD Biosciences). Basal cells were defined as propidium^−^, PE^−^, EpCAM^+^, CD166^mid^, CD49f^hi^ and podoplanin^+^; AT2 cells were defined as propidium^−^, PE^−^, EpCAM^+^, CD166^mid^, CD49f^mid^ and podoplanin^−^ and collected into DNA/RNA shield buffer (Zymo Research). DNA/RNA was extracted using the Quick-DNA/RNA MagBead kit (cat. no. R2130, Zymo Research). After isolation, RRBS libraries were generated, as described in the RRBS methodology and DNA/RNA extraction sections, and RNA-seq was performed^3^. Validation of the purity of the isolated AT2 and basal cells was performed using previously published signatures for the LUAD and LUSC origins, respectively^45^.

### DNA methylation driver discovery

MethSig scores^10^ (https://github.com/HengPan2007/MethSig) were calculated separately for the LUAD and LUSC samples. For each tumor, only the sample with the highest purity was used. Promoter hypermethylation was measured using the differentially hypermethylated cytosine ratio (DHcR), defined as the ratio of hypermethylated cytosines to the total number of profiled CpGs per gene in the promoter region. DMPs were defined based on the counts of methylated and unmethylated loci in tumor versus normal samples using a chi-squared test and 15% FDR. In the normal samples, DHcR ratios were estimated by taking the hypermethylation ratio with respect to the median normal. In the tumor samples, CAMDAC cancer-cell-specific methylation rates were used to calculate the tumor hypermethylation ratios. Genes with no coverage in all samples and no expression in the normal tissue (RSEM counts < 1) were filtered out for subsequent analyses. The expression levels of normal tissue were calculated by averaging RSEM counts across all matched NAT samples. Promoter regions were defined using the default threshold of a ±2-kb window centered on the RefSeq TSS.

MethSig models hypermethylation stochasticity using the PDR^46^ in promoter regions. The PDR measures the proportion of overlapping reads with discordant hypermethylated or hypomethylated CpGs. Applying CAMDAC principles, the cancer-cell-specific tumor PDR (PDR_*t*_) can be expressed as a function of the bulk (PDR_*b*_) and matched normal PDR (PDR_*n*_), weighted by the normal and tumor CN, respectively *n*_*n*_ and *n*_*t*_, and tumor purity (rho; Extended Data Figure 5a).

$${{\mathrm{PDR}}}_{b}\,=\,\frac{{\mathrm{PDR}}_{t}\,{n}_{t}\rho +{{\mathrm{PDR}}}_{n}\,{n}_{n}(1-p)}{{n}_{t}\rho +{n}_{n}(1-p)}{\rm{or}}\; {\rm{equally}}$$

$${{\mathrm{PDR}}}_{t}\,=\,\frac{{{\mathrm{PDR}}}_{b}({n}_{t}\rho +{n}_{n}(1-p))-{{\mathrm{PDR}}}_{n}\,{n}_{n}(1-p)\,}{{n}_{t}\rho \,}\,$$

To validate the application of CAMDAC principles to the methylation stochasticity estimates, we first leveraged SNVs found in genomic regions with loss of heterozygosity (LOH). In these regions, all reads bearing an SNV can be assigned to the tumor cells while all wild-type (WT) reads originate from the normal compartment. A significant correlation was observed between the PDR estimated from CAMDAC and the PDR estimated using SNVs; similarly, a significant correlation was observed between the PDR of NATs and the PDR calculated using WT-LOH (*R* > 0.8, *P* < 2.2 × 10^−17^) (Extended Data Fig. 5b,c).

To evaluate the use of the patient-matched NATs as a representative proxy for the methylation profile of the normal infiltrating cells, we used fluorescence-activated cell sorting (FACS) by DNA content to experimentally separate diploid cell populations from five tumors^17^. As shown in Extended Data Fig. 5d, good agreement was observed between the matched NATs and FACS-purified normal PDRs in all sampled regions (*R* > 0.7). The average PDR per tumor was higher in the CAMDAC cancer-cell-specific estimates than bulk and normal in the vast majority of samples (Extended Data Fig. 5e).

The MethSig functions makeInputMatrix, pvalueBetaReg and pvalueCombine were used to estimate the expected promoter DHcR of tumor samples using a beta regression model and tested against the observed ratio across the cohort.

### Quantification of dosage compensation by DNA methylation

To assess dosage compensation, we calculated the difference in median promoter methylation rates between tumor regions with and without amplification. For instance, a difference of 0.2 between amplified and non-amplified regions indicates that, on average, the allele in half of all amplified tumor regions has become at least 20% more methylated compared to the unamplified regions. In practical terms, for an amplified total CN of five, this could signify that (1) at least one additional promoter copy has become fully methylated in all tumor cells, (2) all copies in all tumor cells have become 20% or more methylated or (3) an additional 20% or more of cells have all copies methylated. The mean gene expression in regions when amplified by SCNAs was compared to when not amplified, with no significant difference being classified as buffered; a significantly lower expression when amplified versus when not amplified was classified as antiscaling based on *t*-test analyses.

### ChIP–seq

For the ChIP–seq analyses, approximately 10^7^ cells from primary cultures derived from the TRACERx samples (two tumor CRUK0977, CRUK0557, and one from NAT CRUK0667 (ref. ^47^)) were fixed with 1% formaldehyde for 10 min in PBS, quenched with 125 mM glycine, washed and lysed; chromatin was sonicated using a Bioruptor Pico (Diagenode), to an average size of 200–700 bp. Immunoprecipitation was performed using 10 μg of chromatin and 2.5 μg of H3K4me3 (cat. no. C15410003) and H3K27me3 (cat. no. C15410195) antibodies. After de-crosslinking, the final DNA purification was performed using the GeneJET PCR Purification Kit (cat. no. K0701, Thermo Fisher Scientific) and quantified using the Qubit dsDNA HS Assay Kit. Sequencing libraries were constructed using the NEBNext Ultra II DNA Library Prep Kit for Illumina (New England Biolabs) and sequenced on the Illumina platform using the Nextseq 2000 system, with a loading concentration of 800 pM and 2% PhiX spike-in, obtaining a total of 500 million reads on average. The reads from the ChIP–seq data were trimmed using TrimGalore v.0.6.6 and aligned to the hg38 genome assembly using Bowtie 2 v.2.4.5. The BAM files were visualized using the interactive tools SeqMonk v.1.48.1 and Integrative Genomics Viewer v.3.2.4. Signals from the histone signal marks were illustrated using BioRender (publication license no. ZG27ZVCQE2).

### Development of the AllChAT pipeline using the EpiATLAS data

We developed the AllChAT pipeline using the EpiATLAS dataset^24^ consisting of 137 samples from five tissue types: bone marrow; brain; colon; kidney; and venous blood. These samples include both tumor and normal tissues, profiled using whole-genome bisulfite sequencing (WGBS) for DNA methylation and chromatin immunoprecipitation for histone modification marks: the activating H3K4me3 and the repressive H3K27me3.

To identify CN aberrations (CNAs) from DNA methylation data, we took the WGBS data from 54 International Human Epigenome Consortium tumors aligned with gemBS and applied the Control-FREEC (v.11.6b) algorithm (window = 50 kb, threshold = 0.8). For a subset of tumors, Control-FREEC was applied to matched whole-genome sequencing and high concordance was observed for WGBS CNAs above 50 Mb in size; therefore, we filtered out all CNAs below this threshold to detect large events and arm-level events. We defined gain and loss CNAs as those greater than or below the ploidy estimate from Control-FREEC, respectively.

We evaluated histone mark intensity, considering their coverage pattern within 2 kb upstream of the TSS of each gene, to determine chromatin accessibility affected by gain or amplification events. Histone marks analyzed included H3K4me3 and H3K27me3. Normalized histone values were obtained by dividing tumor signal averages by normal sample averages, followed by a logarithmic transformation. To identify potential AllChAT oncogene–passenger gene pairs across the genome located in the same amplicon, we used a curated list of 235 known oncogenes and genes located within 20 Mb on the same chromosome, assuming they are under the same CN event.

The pipeline for the identification of AllChAT at pairs of oncogene and passenger gene loci within tumor samples involves: (1) DNA methylation assessment within a gained region. We conducted a one-sided *t*-test to assess whether within a gained or amplified region the differential DNA methylation (tumor versus normal) at the oncogene was lower than that of the passenger. Conversely, in samples where this region is not gained or amplified, we examined whether the differential DNA methylation levels of oncogenes were equal to or more than that of the passenger gene; (2) we next assigned chromatin status using histone mark chromatin immunoprecipitation. In samples with CN gain or amplification, for H3K4me3, a one-sided *t*-test was used to determine whether the tumor/normal differential area under the peak at the TSS of the oncogene was higher than that of the passenger. In samples without CN gain or amplification, we tested whether the tumor/normal differential area under the peak at the TSS of the oncogene was equal to or less than that of the passenger. Alternatively, for H3K27me3, a one-sided *t*-test was used to determine if the tumor/normal differential area under the peak at the TSS of the oncogene was lower than that of the passenger. In samples without CN gain or amplification, we tested whether the tumor/normal differential area under the peak at the TSS of the oncogene was equal to or more than that of the passenger. Loci passing all these criteria were assigned as exhibiting AllChAT.

### Selective enrichment of gene regulatory CpGs using M_R_/M_N_

DNA methylation drivers with potential positive selection in regulatory CpGs were identified using the M_R_/M_N_ metric. To obtain the M_R_/M_N_ ratio per gene, the number of hypermethylation events in all the DMPs covered in every sample and located in gene promoters were considered. Across the cohort, regulatory DMPs were defined as promoter CpGs with differential hypermethylation in tumor versus NAT, with concomitant significantly reduced gene expression using the parametric *t*-test (*P* < 0.05). Nonregulatory DMPs were classified as differentially hypermethylated CpGs not resulting in reduced gene expression. At the gene level, M_R_ represents the number of hypermethylated regulatory promoter CpGs per total number of regulatory promoter CpGs; M_N_ represents the number of hypermethylated nonregulatory promoter CpGs per total number of nonregulatory promoter CpGs; each component was normalized by adding the value of 1 as a pseudocount. Genes without both regulatory and nonregulatory assignments were deemed non-calculable.

The total number of promoter hypermethylation event counts for each regulatory and nonregulatory CpG by gene for LUAD and LUSC are described in Supplementary Table 8. The formula for defining the M_R_/M_N_ ratio per gene was as follows:

$$\frac{{\mathrm{M}_{\mathrm{R}}}}{{\mathrm{M}}_{\mathrm{N}}}=\frac{\frac{{\sum }_{i=1}^{n}{H}_{i}\cdot {R}_{i}+1}{{\sum }_{i=1}^{n}{R}_{i}+1}}{\frac{{\sum }_{i=1}^{n}{H}_{i}\cdot ({1-R}_{i})+1}{n-{\sum }_{i=1}^{n}Ri+1}}$$

where for *i*^*th*^ DMP, i = 1,… *n*, we define its corresponding hypermethylated and regulatory statuses as:

$${H}_{i}=\left\{\begin{array}{ll}1, & if\,{\rm{DMP}}\,is\,hypermethylated,\\ 0, & otherwise\end{array}\right.$$

and

$${R}_{i}=\left\{\begin{array}{ll}1, & if\,{\rm{DMP}}\,is\,regulatory,\\ 0, & if\,{\rm{DMP}}\,is\,nonregulatory\end{array}\right.$$

This ratio was calculated for LUAD and LUSC independently in the TRACERx cohort. Given that DMPs at expression-associated CpGs are more likely to have functional consequences, M_R_/M_N_ ratios greater than 1 imply a selection of regulatory hypermethylation events, while M_R_/M_N_ ratios smaller than 1 imply a selection of nonregulatory hypermethylation events among the total events on DMPs. The impact of M_R_/M_N_ status on gene expression was performed independently in the TCGA cohort. An OR analysis with FDR-adjusted *P* values (*P* < 0.05, *t*-test) was applied to identify significantly affected genes. M_R_/M_N_ has been represented on a logarithmic scale to facilitate interpretation.

### Validation of the M_R_/M_N_ metric

To validate the M_R_/M_N_ metric for the LUAD samples, RRBS and RNA-seq were performed as described in the Methods for 17 regions from ten LUAD tumors, in addition to the adjacent normal tissue. M_R_/M_N_ was calculated as described in the Methods.

To validate whether the DMPs assigned as regulatory and nonregulatory in the discovery cohort maintained these assignments in the validation cohort, we first selected those CpGs associated with a significant expression reduction when hypermethylated compared to tumor regions where they were not hypermethylated (*P* < 0.0001, *t*-test). These CpGs are referred to as ‘significantly regulatory CpGs in the discovery cohort’. Similarly, we selected these CpGs in the discovery cohort with a significant increase in expression in tumor regions when the CpG is hypermethylated versus when it is not (*P* < 0.0001, *t*-test), assigned ‘significantly nonregulatory CpGs in the discovery cohort’.

Next, we assessed the impact of hypermethylation of the selected CpGs in the validation cohort. The ‘significantly regulatory CpGs’ from the discovery cohort were associated with a significant decrease in expression when the CpG was hypermethylated versus when it was not in the validation cohort (*P* = 2.2 × 10^−16^; paired *t*-test; Extended Data Figure 8e). Similarly, the ‘significantly nonregulatory CpGs’ from the discovery cohort were associated with a significant increase in expression when the CpG was hypermethylated versus when it was not in the validation cohort (*P* = 0.0025; paired *t*-test; Extended Data Figure 8e).

### DNA methylation predictions in the TRACERx RNA-seq cohort

To establish a gene expression threshold for the TRACERx RNA-seq cohort that reflects the methylation status of the functional DMPs in the TRACERx RRBS cohort, a bootstrapping methodology was followed. Samples from the TRACERx RRBS cohort with available matched RRBS and RNA-seq data were used. To ensure this metric is robust to multi-region sampling, bootstrapping was performed by randomly selecting a single region per tumor and repeating the process 100 times. Through this process, it was possible to evaluate the mean and 25th, 50th and 75th percentiles of the expression level of genes in tumors when the DMPs were hypermethylated versus when they were not hypermethylated. Next, these values were extrapolated to the gene expression in the TRACERx RNA-seq cohort. For each gene, we dichotomized tumors based on whether or not each promoter DMR was hypermethylated in the RRBS cohort. Hypermethylation-dependent ‘low’ gene expression was assigned in TRACERx RNA-seq samples when gene expression was lower than the 75th percentile (Q3) of expression in the TRACERx RRBS cohort. In contrast, a tumor region was classified as having ‘high’ gene expression if the level was higher than the third quartile of expression in the TRACERx RRBS cohort. At the tumor level, if different tumor regions in the TRACERx RNA-seq cohort exhibited different classifications (for example, R1 with hypermethylation and R2 with hypomethylation), the tumor was classified as having hypermethylation-dependent reduced expression for that gene.

### Survival analysis (TRACERx RNA-seq cohort)

DFS was defined as the period from the date of registration to the time of radiological confirmation of the recurrence of the primary tumor registered for the TRACERx or the time of death by any cause. During the follow-up, three participants with LUAD tumors (CRUK0512, CRUK0428 and CRUK0511) developed a new primary cancer and subsequent recurrence from either the first primary lung cancer or the new primary cancer diagnosed during the follow-up. These cases were censored at the time of the diagnosis of new primary cancer for DFS analysis because of the uncertainty of the origin of the second tumor. As for the participants who harbored synchronous multiple primary lung cancers, when associating genomic and pathological data from the tumors with participant-level clinical information, we used only data from the tumor of the highest pathological TNM stage. Hazard ratios (HRs) and *P* values were calculated using the coxph function of the survival (v.3.4.0) R package, through multivariable Cox regression analyses, adjusted for age, pathological stage, smoking pack-years and receipt of adjuvant therapy. Kaplan–Meier plots were generated using the ggsurvplot function of the survminer (v.0.4.9) R package.

### TIL estimation

TIL scores were estimated using pathological evaluation of regional hematoxylin and eosin-stained slides using established international guidelines, developed by the International Immuno-Oncology Biomarker Working Group, as described in previous reports^48,49^.

### Statistical information

All statistical tests were performed in R (v3.6.3). No statistical methods were used to predetermine the sample sizes of this specific cohort (217 tumors from 59 patients); however, the size of the complete TRACERx cohort at study completion (421 patients) was chosen to provide statistical power for detection of a 0.77 HR effect on the outcome by an ITH variable when split by the median. Tests involving comparisons of distributions were done using a two-tailed Wilcoxon rank-sum test (wilcox.test) unless otherwise specified, using paired or unpaired options where appropriate unless otherwise specified. Tests involving the comparison of groups were done using a two-tailed Fisher’s exact test (fisher.test). HRs and *P* values for the survival analyses were calculated using the survival package. For all statistical tests, the number of data points included are plotted or annotated in the corresponding figure legend.

### Reporting summary

Further information on research design is available in the Nature Portfolio Reporting Summary linked to this article.

## Online content

Any methods, additional references, Nature Portfolio reporting summaries, source data, extended data, supplementary information, acknowledgements, peer review information; details of author contributions and competing interests; and statements of data and code availability are available at 10.1038/s41588-025-02307-x.

## Supplementary information

Supplementary Information Supplementary Figs. 1–7.

Reporting Summary

Supplementary Tables 1–8 Supplementary Table 1: Promoter CpGs in Cluster 1a; Supplementary Table 2: Promoter CpGs in Cluster 1b; Supplementary Table 3: Promoter CpGs in Cluster 2; Supplementary Table 4: Promoter CpGs in Cluster 3; Supplementary Table 5: List of MethSig genes identified in the TRACERx LUAD samples; Supplementary Table 6: List of MethSig genes identified in TRACERx LUSC samples; Supplementary Table 7: AllChAT events in tumor versus normal tissue-derived cells from the TRACERx cohort; Supplementary Table 8: MR/MN value per gene in LUAD and LUSC

## Data Availability

The WES, the RNA-seq and RRBS data (in each case from the TRACERx study) used during this study have been deposited at the European Genome-phenome Archive (EGA), which is hosted by the European Bioinformatics Institute and the Centre for Genomic Regulation under accession nos. EGAS00001006494 (WES), EGAS00001006517 (RNA-seq), EGAS00001006523 (RRBS) and EGAS00001008071 (RBBS and ChIP–seq) and is under controlled access because of its nature and commercial licenses. Specifically, data are available through the CRUK & UCL Cancer Trials Centre (ctc.tracerx@ucl.ac.uk) for academic noncommercial research purposes only and is subject to review of a project proposal by the TRACERx data access committee, entering into an appropriate data access agreement and subject to any applicable ethical approvals. A response to the request for access is typically provided within 10 working days after the committee has received the relevant project proposal and all other required information.

## Code availability

The code used to process the data and generate the figures is available at Zenodo (https://zenodo.org/records/14640157)^50^.
