# Supplementary material for: DNA methylation cooperates with genomic alterations during non-small cell lung cancer evolution
Source: Nat Genet. 2025 Sep 10;57(9):2226–37. doi: 10.1038/s41588-025-02307-x (PMC12425823; doi:10.1038/s41588-025-02307-x)
Supplement: Supplementary file 1 — Supplementary Figs. 1–7. [file 41588_2025_2307_MOESM1_ESM.pdf]

nature genetics

Supplementary information

https://doi.org/10.1038/s41588-025-02307-x

DNA methylation cooperates with genomic alterations during non-small cell lung cancer evolution

In the format provided by the authors and unedited

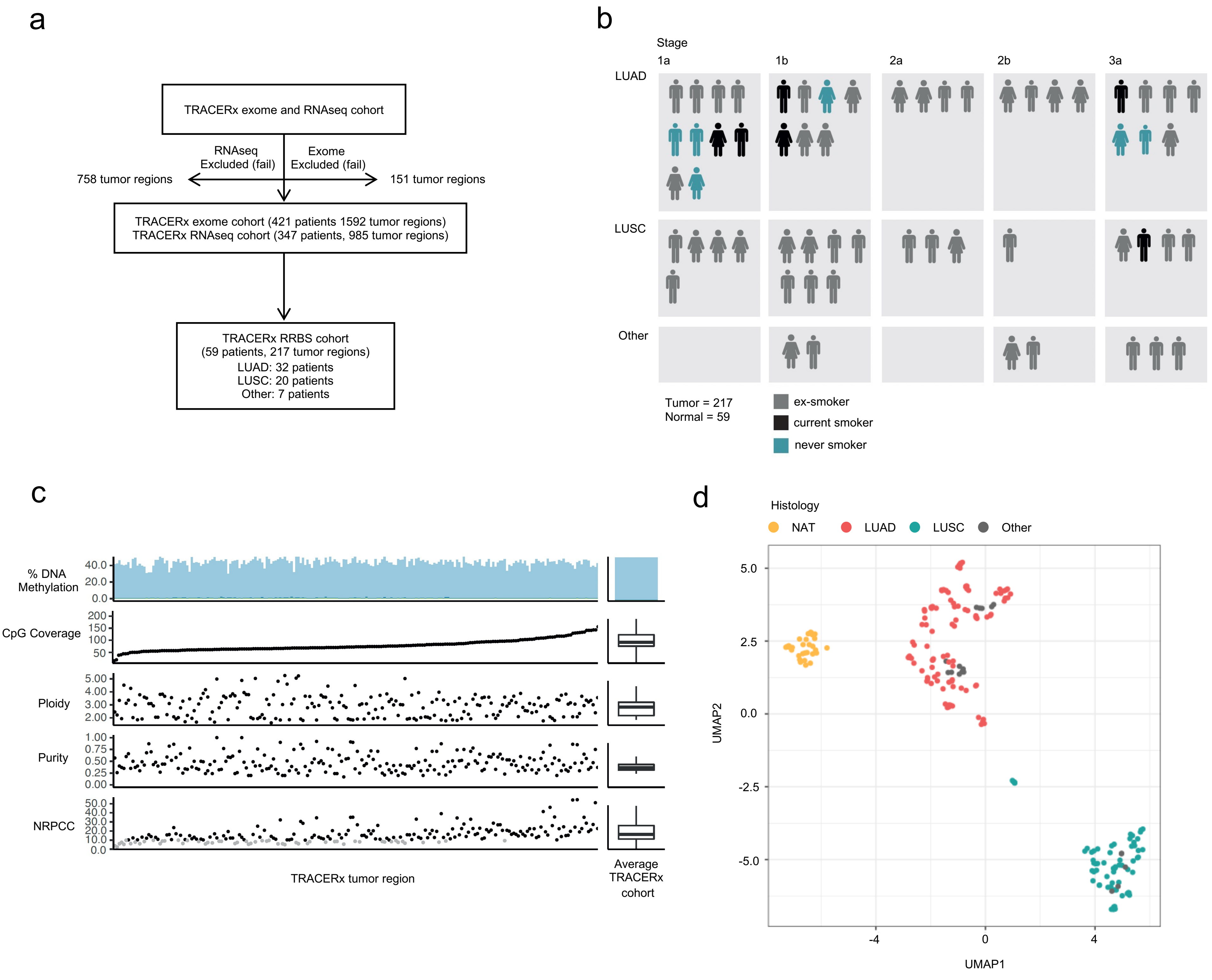

Supplementary Figure 1. Summary of the TRACERx RRBS cohort. a) CONSORT diagram detailing which samples from the TRACERx exome and RNAseq cohort were selected for RRBS analysis. b) Illustration of the patients included in the TRACERx RRBS methylation study classified by histological subtype, clinical stage, sex and smoking status. c) Summary of bulk CpG methylation percentage, coverage, ploidy, purity and number of reads per chromosomal copy (NRPCC) per tumor region. d) UMAP representing the transcriptomic profile of NAT and tumor tissue from samples in the TRACERx cohort.

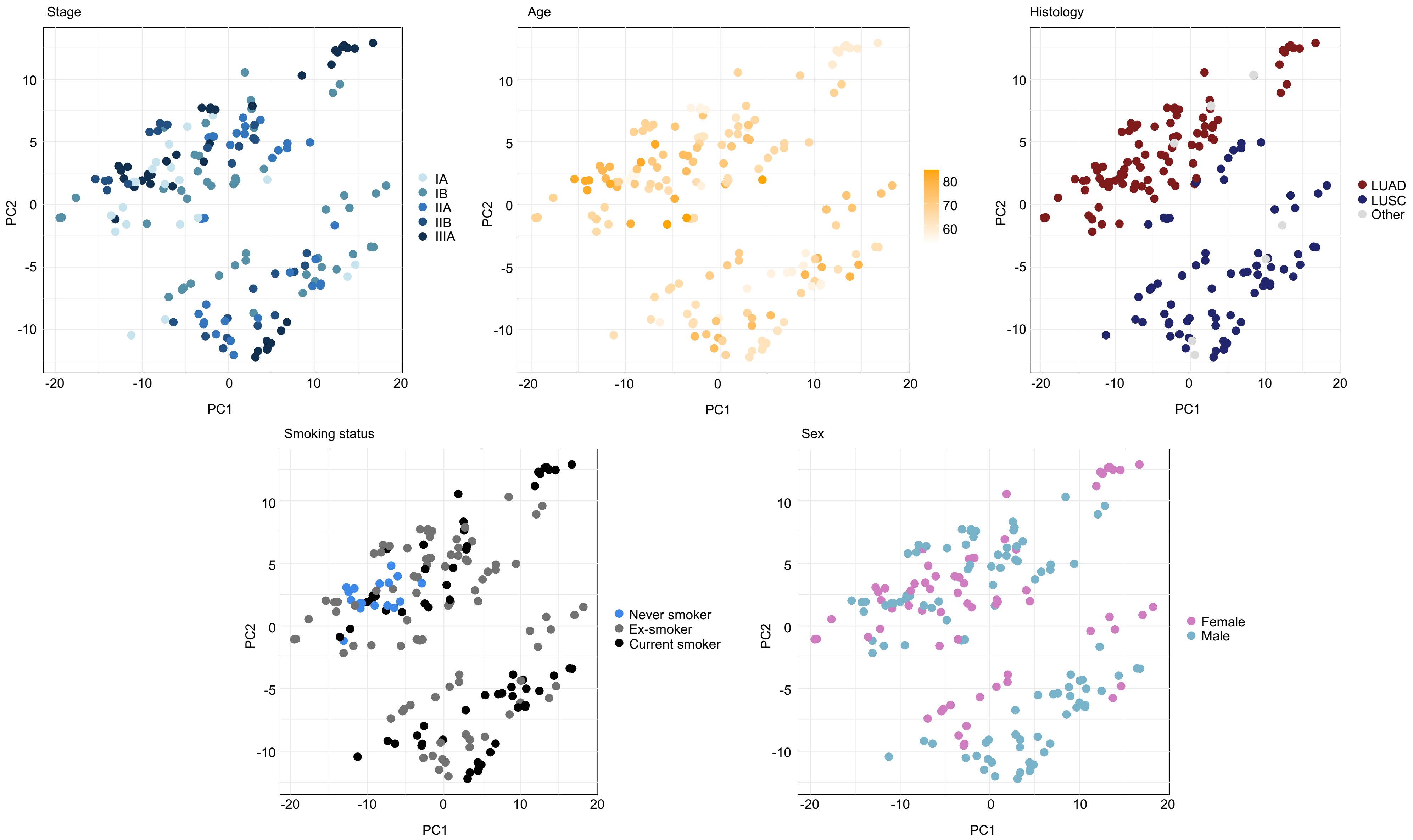

Supplementary Figure 2. UMAP-based representation of TRACERx RRBS cohort stratified by methylation of all CpGs within gene promoters. Clinicopathological characteristics within the cohort are annotated.

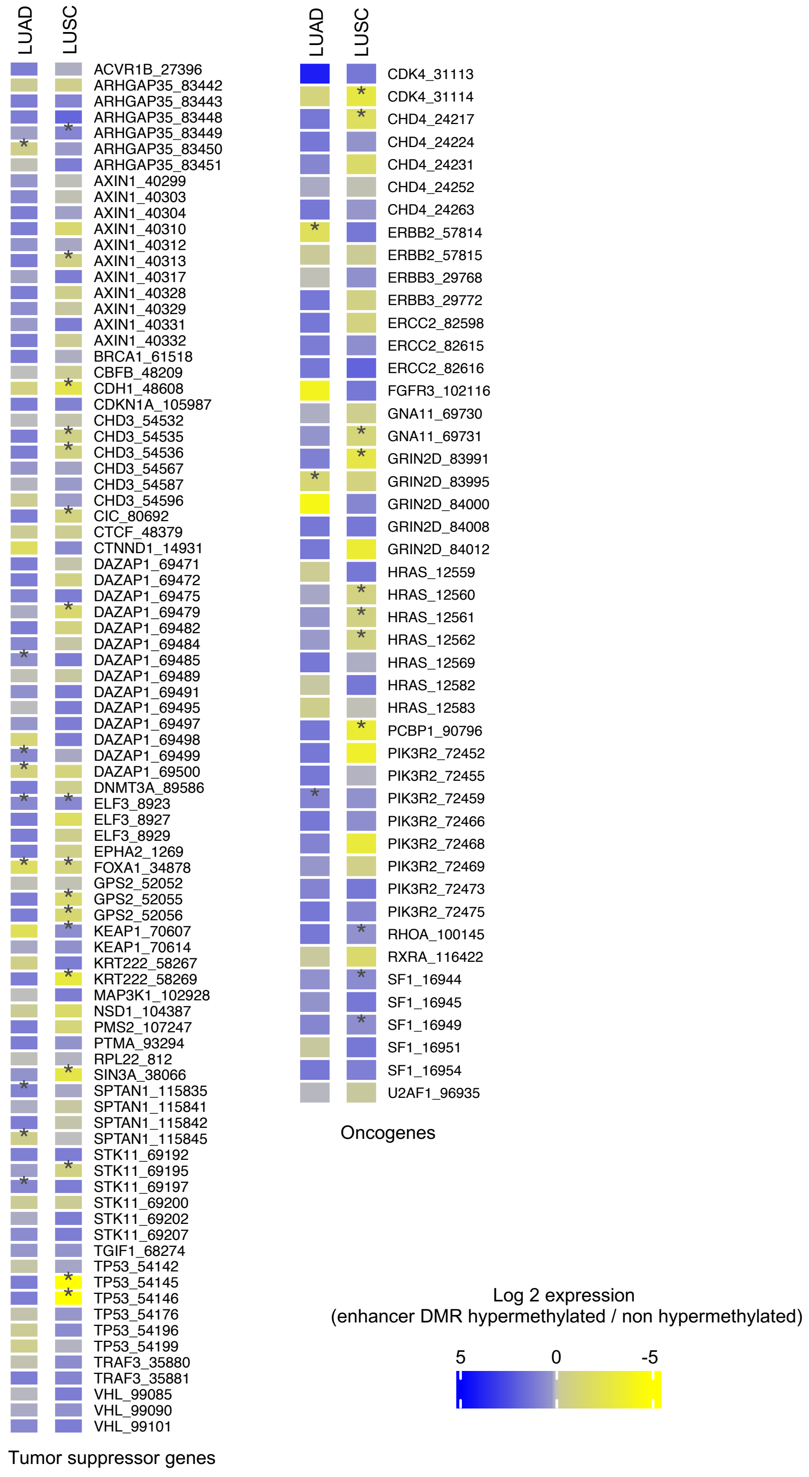

Supplementary Figure 3. Analysis of DNA methylation on genetically annotated enhancers using EnhancerAtlas 2.0. Impact of enhancer methylation on gene expression for genomic TSGs (left) and oncogenes (right) in the TRACERx RRBS cohort for LUAD and LUSC separately. Negative values (yellow) indicate decreased expression in tumors where the enhancer DMR is hypermethylated, while positive values (blue) indicate increased expression in tumors where the enhancer DMR is hypermethylated, with a p-value < 0.05 (*).

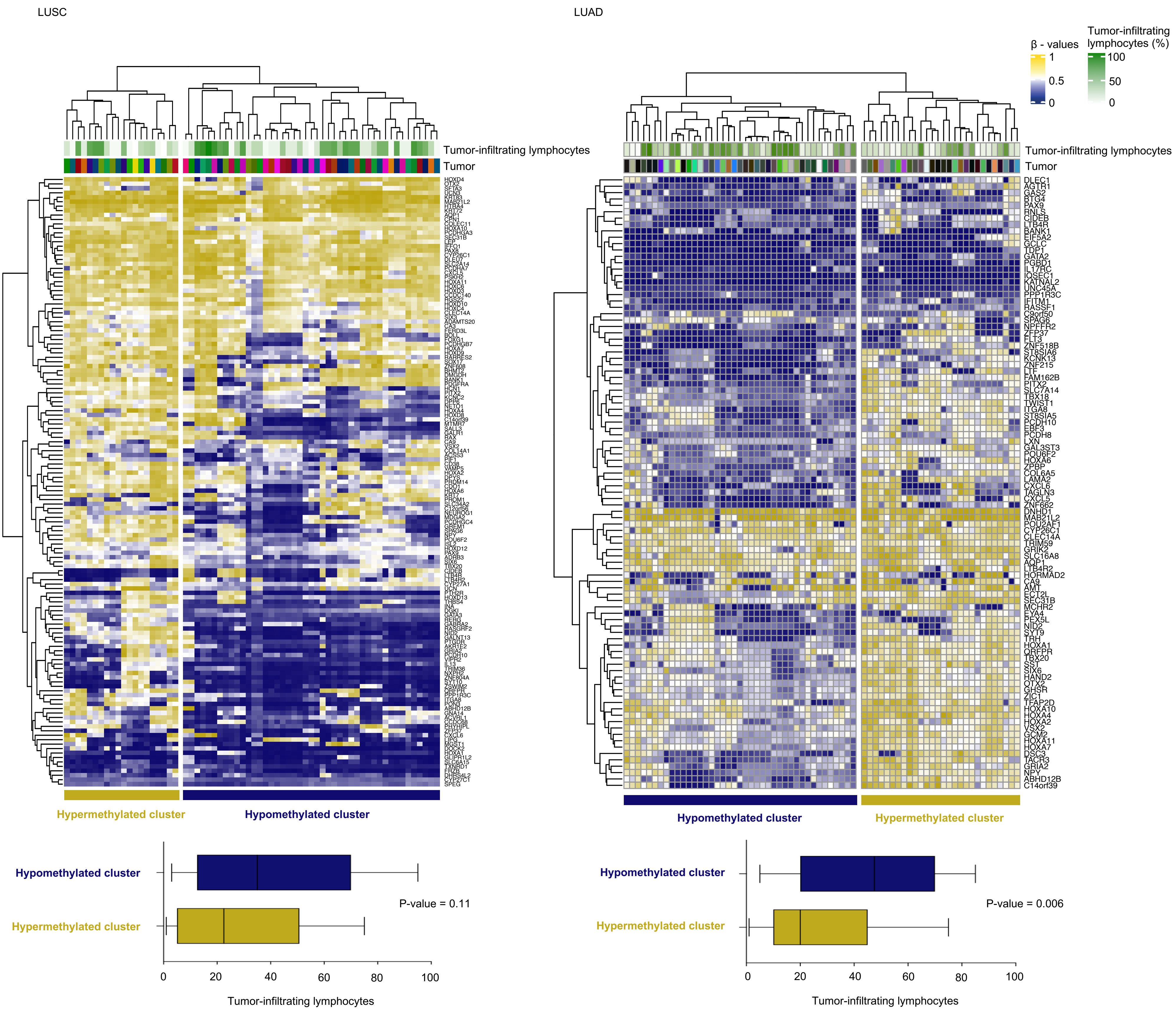

Supplementary Figure 4. Promoter DNA methylation of MethSig genes and association with TILs. Heatmap illustrating the methylation rates of MethSig cancer genes in LUSC (left) and LUAD (right) with clusters representing tumor regions with enriched hypermethylation and hypomethylation of candidate MethSig cancer genes. The box plots quantify the levels of tumor infiltrating lymphocytes (TILs) in each group of samples compared using a Mann-Whitney test (Interquartile ranges, median, and outliers are shown).

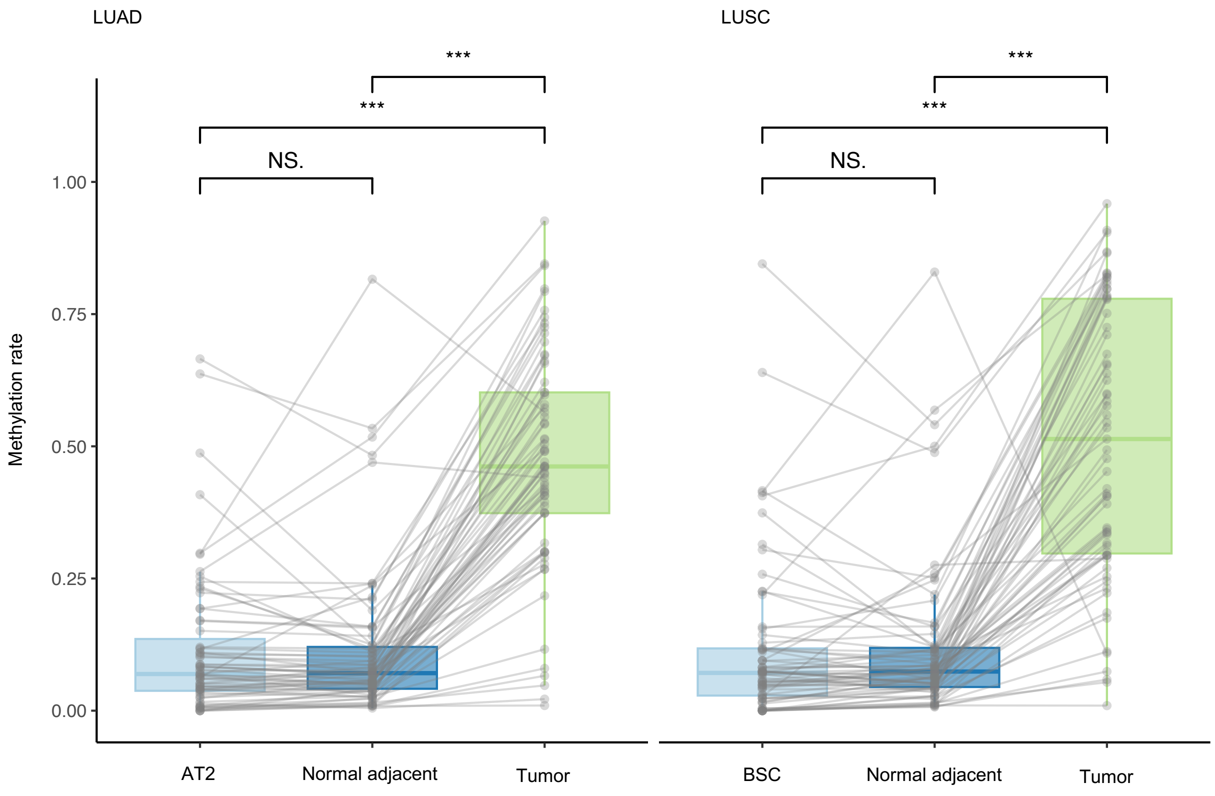

Supplementary Figure 5. Analysis of the methylation rate for LUAD (left) and LUSC (right) candidate MethSig cancer genes comparing the values in the tumor *vs.* Normal adjacent tissue or AT2 for LUAD and BSC for LUSC (ANOVA test, p-value < 0.0001 (***)).

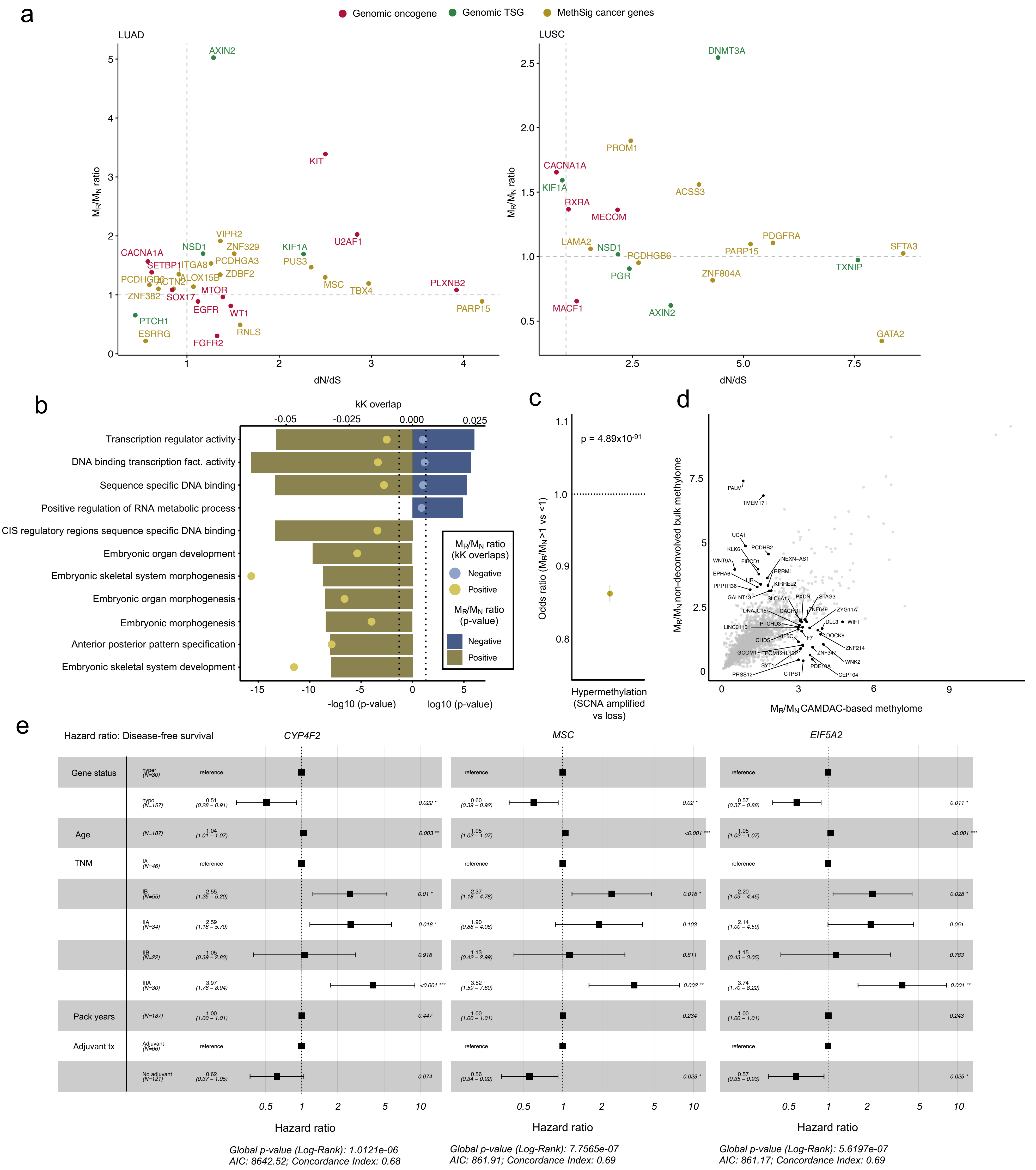

Supplementary Figure 6. Biological characterisation of MethSig cancer genes stratified by MR/MN. a) Comparison between the *dN/dS* ratio based on non-synonymous SNVs and the M_R/M_N ratio of genomic TSGs and candidate MethSig cancer genes in LUAD and LUSC. b) Functional enrichment analysis with GO terms for MethSig cancer genes with $M_R/M_N > 1$ (yellow dot represents overlap proportion and yellow box illustrates p-values) and $M_R/M_N < 1$ (blue dot represents overlap proportion and blue box illustrates p-values) in LUAD. The kK overlap refers to the proportion of MethSig cancer genes associated with each GO term. c) Odds ratio of promoter DNA hypermethylation for genes with $M_R/M_N > 1$ and < 1, located in regions with amplification versus copy number loss in LUAD. d) Cloud plot illustrating the distribution of genes based on their MR/MN values in non-deconvolved bulk methylome (y-axis) and CAMDAC-based methylome (x-axis). Genes with $M_R/M_N > 1$ from either bulk analysis or following CAMDAC deconvolution in LUAD are labeled. e) Multivariate Cox analysis, with 95% confidence intervals, for the three $M_R/M_N > 1$ MethSig cancer genes impacting DFS in the LUAD TRACERx cohort.

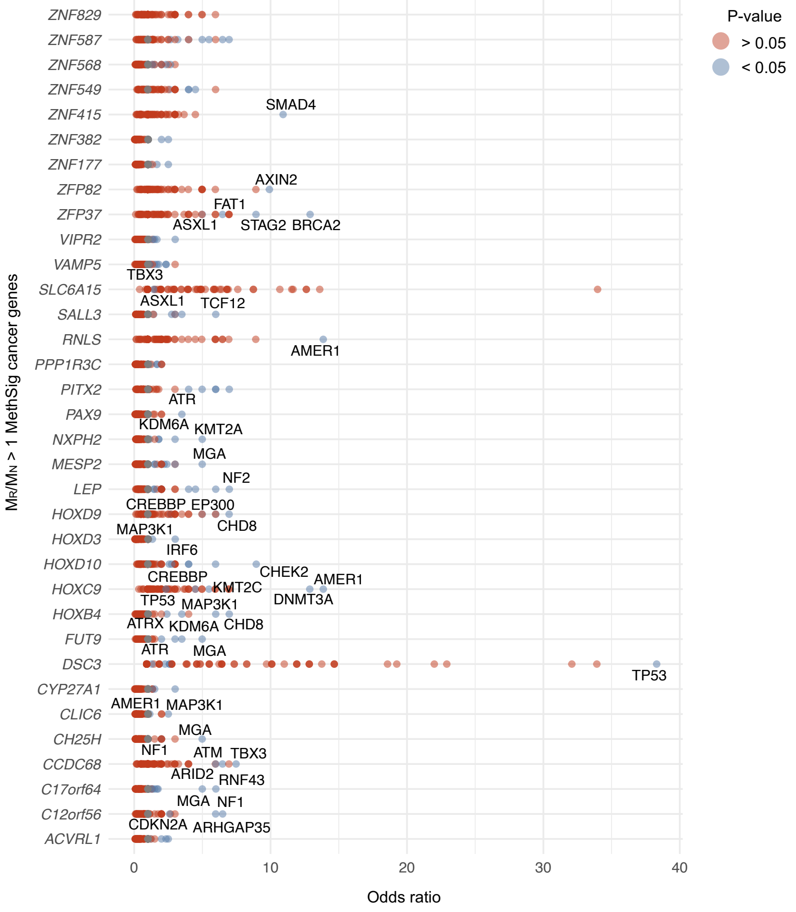

Supplementary Figure 7. Odds ratio highlighting the co-occurrence of promoter DNA hypermethylation events for M_R/M_N>1 MethSig cancer genes and driver mutations in canonical TSGs in LUSC. Significant co-occurrences are labelled.
