# Supplementary material for: DNA methylation cooperates with genomic alterations during non-small cell lung cancer evolution
Source: Nat Genet. 2025 Sep 10;57(9):2226–37. doi: 10.1038/s41588-025-02307-x (PMC12425823; doi:10.1038/s41588-025-02307-x)
Supplement: Supplementary file 2 — Reporting Summary [file 41588_2025_2307_MOESM2_ESM.pdf]

nature portfolio

Corresponding author(s): Peter Van Loo
Nnennaya Kanu

Last updated by author(s): 2025/03/12

Reporting Summary

Nature Portfolio wishes to improve the reproducibility of the work that we publish. This form provides structure for consistency and transparency in reporting. For further information on Nature Portfolio policies, see our Editorial Policies and the Editorial Policy Checklist.

Statistics

For all statistical analyses, confirm that the following items are present in the figure legend, table legend, main text, or Methods section.

n/a	Confirmed	
☐	☒	The exact sample size (n) for each experimental group/condition, given as a discrete number and unit of measurement
☐	☒	A statement on whether measurements were taken from distinct samples or whether the same sample was measured repeatedly
☐	☒	The statistical test(s) used AND whether they are one- or two-sided<br>*Only common tests should be described solely by name; describe more complex techniques in the Methods section.*
☐	☒	A description of all covariates tested
☐	☒	A description of any assumptions or corrections, such as tests of normality and adjustment for multiple comparisons
☐	☒	A full description of the statistical parameters including central tendency (e.g. means) or other basic estimates (e.g. regression coefficient) AND variation (e.g. standard deviation) or associated estimates of uncertainty (e.g. confidence intervals)
☐	☒	For null hypothesis testing, the test statistic (e.g. F, t, r) with confidence intervals, effect sizes, degrees of freedom and P value noted<br>*Give P values as exact values whenever suitable.*
☒	☐	For Bayesian analysis, information on the choice of priors and Markov chain Monte Carlo settings
☒	☐	For hierarchical and complex designs, identification of the appropriate level for tests and full reporting of outcomes
☐	☒	Estimates of effect sizes (e.g. Cohen's d, Pearson's r), indicating how they were calculated

Our web collection on statistics for biologists contains articles on many of the points above.

Software and code

Policy information about availability of computer code

Data collection	No software was used to collect data
Data analysis	R (version 3.6.3)<br><br>Alignment and QC:<br>FastQC (version 0.11.8)<br>FastQ Screen (version 0.13.0)<br>bwa-mem (version 0.7.17)<br>Sambamba (version 0.7.0)<br>Picard Tools (version 2.21.9)<br>GATK (version 3.8.1)<br>Somalier (version 0.2.7)<br>Samtools (version 1.9)<br>Conpair (version 0.2)<br>Bismark (version 0.23.0)<br>Bowtie2 (version 2.4.2)<br><br>Variant Calling:<br>SAMtools (version 1.10)<br>VarScan2 (version 2.4.4)<br>MuTect (version 1.1.7)

bam-readcount (version 0.7.4)
Annovar (version: Revision 529)

Heterozygous single nucleotide polymorphism (SNP) identification:
Platypus (version 0.8.1)

Somatic Copy Number aberration detection:
VarScan2 (version 2.4.4)
ASCAT (version 2.3)
Sequenza (version 2.1.2)

R packages used in version 3.6.3:
fst (version 0.9.4)
tidyverse (version 1.3.0)
survival (version 3.4)
ggplot2 (version 3.3.2)
dplyr (version 1.0.2)
tidyr (version 1.1.2)
gridExtra (version 2.3)
cowplot (version 1.1.0)
survminer (version 0.4.9)
survival (version 3.4.0)
ggpubr (version 0.4.0)
ggalluvial (version 0.12.3)
gtsummary (version 1.5.0)
reshape2 (version 1.4.4)
tibble (version 3.0.4)
gtable (version 0.3.0)
RColorBrewer (version 1.1-2)
plyr (version 1.8.6)
dndscv (version 0.0.1.0)
deconstructSigs (version 1.9.0)
ggrepel (version 0.8.2)
GenomicRanges (version 1.38.0)
rlist (version 0.4.6.2)
tidytext (version 0.2.3)
stringr (version 1.4.0)
magick (version 2.7.3)
data.table (version 1.13.2)
DiagrammR (version 1.0.1)
magrittr (version 2.0.1)
ComplexHeatmap (version 2.4.5)
Biorender (License: ZG27ZVCQE2)
The reads from ChIP-seq data were trimmed using Trim Galore (Version 0.6.6) and aligned to the hg38 genome assembly using Bowtie2v2.4.5.
The bam files were visualised using the interactive tools SeqMonk (Version 1.48.1) and IGV (Version 3.2.4)

All code to reproduce the figures will be available.

For manuscripts utilizing custom algorithms or software that are central to the research but not yet described in published literature, software must be made available to editors and reviewers. We strongly encourage code deposition in a community repository (e.g. GitHub). See the Nature Portfolio guidelines for submitting code & software for further information.

Data

Policy information about availability of data

All manuscripts must include a data availability statement. This statement should provide the following information, where applicable:

- Accession codes, unique identifiers, or web links for publicly available datasets
- A description of any restrictions on data availability
- For clinical datasets or third party data, please ensure that the statement adheres to our policy

The Whole exome sequencing (WES) data, the RNA sequencing (RNA seq) data and the Reduced representation bisulfite sequencing (RRBS) data (in each case from the TRACERx study) used during this study have been deposited at the European Genome–phenome Archive (EGA), which is hosted by The European Bioinformatics Institute (EBI) and the Centre for Genomic Regulation (CRG) under the accession codes EGAS00001006494 (WES), EGAS00001006517 (RNAseq) and EGAS00001006523 and EGAS00001008071 (RBBS and ChipSEQ); access is controlled by the TRACERx data access committee. Details on how to apply for access are available at the linked page.

Research involving human participants, their data, or biological material

Policy information about studies with human participants or human data. See also policy information about sex, gender (identity/presentation), and sexual orientation and race, ethnicity and racism.

Reporting on sex and gender	The effects of sex and/or gender have not been considered in the recruitment of patients. No differences have been observed between men and women in patient recruitment, and there are no differences in any of our analyses.
Reporting on race, ethnicity, or other socially relevant groupings	*Please specify the socially constructed or socially relevant categorization variable(s) used in your manuscript and explain why they were used. Please note that such variables should not be used as proxies for other socially constructed/relevant variables (for example, race or ethnicity should not be used as a proxy for socioeconomic status).*<br>*Provide clear definitions of the relevant terms used, how they were provided (by the participants/respondents, the researchers, or third parties), and the method(s) used to classify people into the different categories (e.g. self-report, census or administrative data, social media data, etc.)*<br>*Please provide details about how you controlled for confounding variables in your analyses.*
Population characteristics	421 patients are included in this TRACERx cohort. 44.6% are females , 55.4% males; 93% are smokers of have a smoking history, 7% are never smokers; 25% of patients were diagnosed at stage IA, 25% at IB, 17.8% at IIA, 13.5% at IIB, 18.5% at IIIA and 0.2% at IIIB; 52% of diagnosed tumours were adenocarcinomas, 28.8% were squamous cell carcinomas and 19.2% were of other histological subtypes; 93% of the cohort is from a white ethnic background and the mean age of the patients is 69, ranging between 34 and 92.<br><br>Please note that the study started recruiting patients in 2016, when TNM version 7 was standard of care. The up-to-date inclusion/exclusion criteria now utilizes TNM version 8.<br><br>TRACERx inclusion and exclusion criteria<br><br>Inclusion Criteria:<br>_Written Informed consent<br>_Patients ≥18 years of age, with early stage I-IIIB disease (according to TNM 8th edition) who are eligible for primary surgery.<br>_Histopathologically confirmed NSCLC, or a strong suspicion of cancer on lung imaging necessitating surgery (e.g. diagnosis determined from frozen section in theatre)<br>_Primary surgery in keeping with NICE guidelines planned<br>_Agreement to be followed up at a TRACERx site<br>_Performance status 0 or 1<br>_Minimum tumor diameter at least 15mm to allow for sampling of at least two tumour regions (if 15mm, a high likelihood of nodal involvement on pre-operative imaging required to meet eligibility according to stage, i.e. T1N1-3)<br><br>Exclusion Criteria:<br>_Any other* malignancy diagnosed or relapsed at any time, which is currently being treated (including by hormonal therapy).<br>_Any other* current malignancy or malignancy diagnosed or relapsed within the past 3 years**.<br>*Exceptions are: non-melanomatous skin cancer, stage 0 melanoma in situ, and in situ cervical cancer<br>**An exception will be made for malignancies diagnosed or relapsed more than 2, but less than 3, years ago only if a pre-operative biopsy of the lung lesion has confirmed a diagnosis of NSCLC.<br>_Psychological condition that would preclude informed consent<br>_Treatment with neo-adjuvant therapy for current lung malignancy deemed necessary<br>_Post-surgery stage IV<br>_Known Human Immunodeficiency Virus (HIV), Hepatitis B Virus (HBV), Hepatitis C Virus (HCV) or syphilis infection.<br>_Sufficient tissue, i.e. a minimum of two tumor regions, is unlikely to be obtained for the study based on pre-operative imaging<br><br>Patient ineligibility following registration<br>_There is insufficient tissue<br>_The patient is unable to comply with protocol requirements<br>_There is a change in histology from NSCLC following surgery, or NSCLC is not confirmed during or after surgery.<br>_Change in staging to IIIC or IV following surgery<br>_The operative criteria are not met (e.g. incomplete resection with macroscopic residual tumors (R2)). Patients with microscopic residual tumors (R1) are eligible and should remain in the study<br>_Adjuvant therapy other than platinum-based chemotherapy and/or radiotherapy is administered.
Recruitment	When patients are initially diagnosed with stage I-III lung cancer and then referred for surgical resection, a research nurse identifies them on a clinic/operating list. The patient has an initial eligibility assessment and then provided with written information about the TRACERx study and he/she can ask the research nurse any questions.<br><br>Patients have to agree to provide serial blood samples whenever they attend clinic for routine blood sampling, so this represents the only main potential self-selecting bias (i.e. only patients willing to do this would participate). However, it is unclear how this would affect the biomarker analyses. Also, the gender and ethnicity characteristics are in line with patients seen in routine practice.<br><br>Inclusion and exclusion criteria are summarised above.
Ethics oversight	The study was approved by the NRES Committee London with the following details:<br>Study title: TRAcking non small cell lung Cancer Evolution through therapy (Rx)

REC reference: 13/LO/1546
Protocol number: UCL/12/0279
IRAS project ID: 138871

Note that full information on the approval of the study protocol must also be provided in the manuscript.

Field-specific reporting

Please select the one below that is the best fit for your research. If you are not sure, read the appropriate sections before making your selection.

☒ Life sciences ☐ Behavioural & social sciences ☐ Ecological, evolutionary & environmental sciences

For a reference copy of the document with all sections, see nature.com/documents/nr-reporting-summary-flat.pdf

Life sciences study design

All studies must disclose on these points even when the disclosure is negative.

Sample size	No statistical methods were used to predetermine sample size. The sample size of 59 patients (217 tumour regions) that passed quality check filters for RRBS included 32 LUAD, 20 LUSC, and 7 other NSCLC subtypes, all with matched normal adjacent tissue (NAT). Among these, 31 were stage I, 14 stage II, and 14 stage III. In terms of smoking history, 47 were former smokers, 6 were current smokers, and 6 were never smokers.
Data exclusions	Please see study inclusion/exclusion criteria below. Additionally, samples which fail quality control metrics including low tumor purity (<10%) were also excluded from analysis.
Replication	TRACERx is a prospective longitudinal study. As such, the results shown here are not the result of an experimental set up. This is the half-way point of the TRACERx study and reflects hypothesis generating analysis.
Randomization	Randomization is not relevant as this is an observational study.
Blinding	Blinding is not relevant as this is an observational study. Patients were not allocated to any intervention and they were followed up and assessed as per routine practice. No biomarker results (tissue and bloods) are reported back to patients, so there is no likelihood of people changing their behaviours based on these findings. The laboratory analyses were all performed without knowing the outcome (DFS or survival) status of the patients, which represents a form of blinding.

Reporting for specific materials, systems and methods

We require information from authors about some types of materials, experimental systems and methods used in many studies. Here, indicate whether each material, system or method listed is relevant to your study. If you are not sure if a list item applies to your research, read the appropriate section before selecting a response.

Materials & experimental systems

n/a	Involved in the study	
☐	☒	Antibodies
☐	☒	Eukaryotic cell lines
☒	☐	Palaeontology and archaeology
☒	☐	Animals and other organisms
☐	☒	Clinical data
☒	☐	Dual use research of concern
☒	☐	Plants

Methods

n/a	Involved in the study	
☐	☒	ChIP-seq
☒	☐	Flow cytometry
☒	☐	MRI-based neuroimaging

Antibodies

Antibodies used	Immunoprecipitation was performed using 10ug of chromatin and 2.5 ug of H3K4me3 (C15410003) and H3K27me3 (C15410195) antibodies. Isolated cells were blocked with anti-Fc block (Fc1, BD) and stained with the following antibodies using a standar concentration of 0.25 µg/106 cells: CD45-PE (HI30, BDbioscience), CD235a-PE (HIR2, BDbioscience), CD140b-PE (28D4, BDbioscience), CD31-PE (WM59, BDbioscience), EpCAM-FITC (VU-1D9, STEMCELL tech.), podoplanin-APC-Cy7 (NC-08, BioLegend), CD166-APC (eBioALC48, ThermoFisher), CD49f-PE-Cy7 (GoH3, ThermoFisher). Basal cells were defined as propidium-, PE-, EpCAM+, CD166mid, CD49fhi and podoplanin+; alveolar type II cells were defined as propidium-, PE-, EpCAM+, CD166mid, CD49fmid and podoplanin-
Validation	The protocol for the isolation and identification of both basal cells and type II alveolar cells has been previously described in Weeden, C. E. et al. Lung Basal Stem Cells Rapidly Repair DNA Damage Using the Error-Prone Nonhomologous End-Joining Pathway. PLoS Biol. 15, 1–27 (2017). The H3K4me3 and H3K27me3 antibodies from the commercial company Diagenode have been previously cited in Sipola, J. Plasma Cell-Free DNA Chromatin Immunoprecipitation Profiling Depicts Phenotypic and Clinical Heterogeneity in Advanced

Prostate Cancer. Cancer Res. 2025 Feb 17;85(4):791-807

Eukaryotic cell lines

Policy information about cell lines and Sex and Gender in Research

Cell line source(s): Three primary cell lines derived from TRACERx study patients previously reported have been used

Authentication: *Describe the authentication procedures for each cell line used OR declare that none of the cell lines used were authenticated.*

Mycoplasma contamination: *Confirm that all cell lines tested negative for mycoplasma contamination OR describe the results of the testing for mycoplasma contamination OR declare that the cell lines were not tested for mycoplasma contamination.*

Commonly misidentified lines (See ICLAC register): *Name any commonly misidentified cell lines used in the study and provide a rationale for their use.*

Clinical data

Policy information about clinical studies

All manuscripts should comply with the ICMJE guidelines for publication of clinical research and a completed CONSORT checklist must be included with all submissions.

Clinical trial registration: TRACERx Lung https://clinicaltrials.gov/ct2/show/NCT01888601, approved by an independent Research Ethics Committee, 13/LO/1546

Study protocol: https://clinicaltrials.gov/ct2/show/NCT01888601

Data collection: Clinical and pathological data is collected from patients during study follow up - this period is a minimum of five years. Data collection is overseen by the sponsor of the study (Cancer Research UK & UCL Cancer Trials Centre) and takes place in hospitals across the United Kingdom. A centralised database called MACRO is used for this purpose. Recruitment started in April 2014 and is still ongoing (in London and Manchester).

Outcomes: The main clinical outcomes is:
Disease-free survival (DFS) – measured from the time of study registration to date of first lung recurrence or death from any cause. Patients who do not have these events are censored at the date last known to be alive (including patients who developed a new primary tumour that has been shown biologically to not be linked to the initial primary lung tumour).

Plants

Seed stocks: *Report on the source of all seed stocks or other plant material used. If applicable, state the seed stock centre and catalogue number. If plant specimens were collected from the field, describe the collection location, date and sampling procedures.*

Novel plant genotypes: *Describe the methods by which all novel plant genotypes were produced. This includes those generated by transgenic approaches, gene editing, chemical/radiation-based mutagenesis and hybridization. For transgenic lines, describe the transformation method, the number of independent lines analyzed and the generation upon which experiments were performed. For gene-edited lines, describe the editor used, the endogenous sequence targeted for editing, the targeting guide RNA sequence (if applicable) and how the editor was applied.*

Authentication: *Describe any authentication procedures for each seed stock used or novel genotype generated. Describe any experiments used to assess the effect of a mutation and, where applicable, how potential secondary effects (e.g. second site T-DNA insertions, mosiacism, off-target gene editing) were examined.*

ChIP-seq

Data deposition

☒ Confirm that both raw and final processed data have been deposited in a public database such as GEO.

☒ Confirm that you have deposited or provided access to graph files (e.g. BED files) for the called peaks.

Data access links (*May remain private before publication.*): EGAS00001008071

Files in database submission: EGAF00007612978;EGAF00007612979;EGAF00007612980;EGAF00007612981;EGAF00007612982;EGAF00007612983;EGAF00007612984;EGAF00007612985;EGAF00007612986;EGAF00007612987;EGAF00007612988;EGAF00007612989;EGAF00007612990;EGAF00007612991;EGAF00007612992;EGAF00007612993;EGAF00007612994;EGAF00007612995;EGAF00007612996;EGAF00007612997;EGAF00007612998;EGAF00007612999;EGAF00007613000;EGAF00007613001;EGAF00007613002;EGAF00007613003;EGAF00007613004;EGAF00007613005;EGAF00007613006;EGAF00007613007;EGAF00007613008;EGAF00007613009;EGAF00007613010;EGAF00007613011;EGAF00007613012;EGAF00007613013;EGAF00007613014;EGAF00007613015;EGAF00007613016;EGAF00007613017;EGAF00007613018;EGAF00007613019;EGAF00007613020;EGAF00007613021;EGAF00007613022;EGAF00007613023;EGAF00007613024;EGAF00007613025;EGAF00007613026;EGAF00007613027;EGAF00007613028;EGAF00007613029;EGAF00007613030;EGAF00007613031;EGAF00007613032;EGAF00007613033;EGAF00007613

034;EGAF00007613035;EGAF00007613036;EGAF00007613037;EGAF00007613038;EGAF00007613039;EGAF00007613040;EGAF00007613041;EGAF00007613042;EGAF00007613043;EGAF00007613044;EGAF00007613045;EGAF00007613046;EGAF00007613047;EGAF00007613048;EGAF00007613049;EGAF00007613050;EGAF00007613051;EGAF00007613052;EGAF00007613053;EGAF00007613054;EGAF00007613055;EGAF00007613056;EGAF00007613057;EGAF00007613058;EGAF00007613059;EGAF00007613060;EGAF00007613061;EGAF00007613062;EGAF00007613063;EGAF00007613064;EGAF00007613065;EGAF00007613066;EGAF00007613067;EGAF00007613068;EGAF00007613069;EGAF00007613070;EGAF00007613071;EGAF00007613072;EGAF00007613073;EGAF00007613074;EGAF00007613075;EGAF00007613076;EGAF00007613077;EGAF00007613078;EGAF00007613079;EGAF00007613080;EGAF00007613081;EGAF00007613082;EGAF00007613083;EGAF00007613084;EGAF00007613085;EGAF00007613086;EGAF00007613087;EGAF00007613088;EGAF00007613089;EGAF00007613090;EGAF00007613091;EGAF00007613092;EGAF00007613093;EGAF00007613096;EGAF00007613097;EGAF00007613098;EGAF00007613099;EGAF00007613100;EGAF00007613101;EGAF00007613102;EGAF00007613103;EGAF00007613104;EGAF00007613105;EGAF00007613106;EGAF00007613107;EGAF00007613108;EGAF00007613109;EGAF00007613110;EGAF00007613111;EGAF00007613112;EGAF00007613113;EGAF00007613114;EGAF00007613115

Genome browser session (e.g. UCSC)

not longer applicable

Methodology

Replicates

Three replicates por IP

Sequencing depth

After de-crosslinking, the final DNA purification was performed using the GeneJET PCR Purification Kit (Thermo Scientific, catalogue number K0701) and quantified using Qubit dsDNA HS Assay Kit (Thermo Fisher Scientific). Sequencing libraries were constructed using the NEBNext Ultra II DNA Library Prep Kit for Illumina (NEB) and sequenced on the Illumina platform using Nextseq2000, with a loading concentration of 800pM and 2% PhiX spike-in and obtaining a total of 500 million reads on average. The reads from ChIP-seq data were trimmed using Trim Galore and aligned to the hg38 genome assembly using Bowtie2v2.4.5. The bam files were visualised using the interactive tools SeqMonk and IGV. The histone signal was illustrated using BioRender.

Antibodies

We have used the commercial antibodies H3K4me3 (C15410003) and H3K27me3 (C15410195).

Peak calling parameters

The reads from ChIP-seq data were trimmed using Trim Galore and aligned to the hg38 genome assembly using Bowtie2v2.4.5.

Data quality

The bam files were evaluated using the interactive tool SeqMonk

Software

The reads from ChIP-seq data were trimmed using Trim Galore and aligned to the hg38 genome assembly using Bowtie2v2.4.5. The bam files were visualised using the interactive tools SeqMonk and IGV.
